# Cytoplasmic Male Sterility-Associated Chimeric Open Reading Frames Identified by Mitochondrial Genome Sequencing of Four *Cajanus* Genotypes

**DOI:** 10.1093/dnares/dst025

**Published:** 2013-06-20

**Authors:** Reetu Tuteja, Rachit K. Saxena, Jaime Davila, Trushar Shah, Wenbin Chen, Yong-Li Xiao, Guangyi Fan, K. B. Saxena, Andrew J. Alverson, Charles Spillane, Christopher Town, Rajeev K. Varshney

**Affiliations:** 1 International Crops Research Institute for the Semi-Arid Tropics (ICRISAT), Patancheru, India; 2 Plant and AgriBiosciences Centre (PABC), School of Natural Sciences, National University of Ireland Galway, Galway, Ireland; 3 Center for Plant Science Innovation, University of Nebraska, Lincoln, USA; 4 Division of Biomedical Statistics and Informatics, Mayo Clinic, Rochester, USA; 5 Beijing Genomics Institute (BGI)-Shenzhen, Shenzhen, China; 6 J. Craig Venter Institute (JCVI), Rockville, USA; 7 Department of Biological Sciences, University of Arkansas, Arkansas, USA

**Keywords:** mitochondria, pigeonpea, next-generation sequencing, cytoplasmic male sterility, open reading frames

## Abstract

The hybrid pigeonpea (*Cajanus cajan*) breeding technology based on cytoplasmic male sterility (CMS) is currently unique among legumes and displays major potential for yield increase. CMS is defined as a condition in which a plant is unable to produce functional pollen grains. The novel chimeric open reading frames (ORFs) produced as a results of mitochondrial genome rearrangements are considered to be the main cause of CMS. To identify these CMS-related ORFs in pigeonpea, we sequenced the mitochondrial genomes of three *C. cajan* lines (the male-sterile line ICPA 2039, the maintainer line ICPB 2039, and the hybrid line ICPH 2433) and of the wild relative (*Cajanus cajanifolius* ICPW 29). A single, circular-mapping molecule of length 545.7 kb was assembled and annotated for the ICPA 2039 line. Sequence annotation predicted 51 genes, including 34 protein-coding and 17 RNA genes. Comparison of the mitochondrial genomes from different *Cajanus* genotypes identified 31 ORFs, which differ between lines within which CMS is present or absent. Among these chimeric ORFs, 13 were identified by comparison of the related male-sterile and maintainer lines. These ORFs display features that are known to trigger CMS in other plant species and to represent the most promising candidates for CMS-related mitochondrial rearrangements in pigeonpea.

## 1. Introduction

Angiosperm mitochondrial genomes are unique in eukaryotes because of their high rates of rearrangement, sequence duplication, ongoing gene loss, and frequent incorporation of foreign DNA.^1–3^ Land plant mitochondrial genomes vary in size from 105^4^ to >11 000 kb.^5^ Hence, the smallest land plant mitochondrial genome (*Physcomitrella patens*, 105 kb) is still ∼11 times larger than the human mitochondrial genome^6^ (∼16 kb). Several studies have reported the presence of subgenomic circles in mitochondrial genomes that have arisen from recombination events.^7,8^ While such recombination events in plant mitochondria increase the complexity of their genome structures, recombination has also been proposed to maintain genomic stability and may also provide a mechanism to increase genetic variation in the absence of sexual reproduction.^9,10^

Rearrangements in mitochondrial genomes are of considerable biotechnological interest as they can cause cytoplasmic male sterility (CMS), which is a valuable tool for plant breeding programmes. Male sterility is caused by the failure of a plant to produce functional pollen grains.^11^ CMS is a maternally inherited trait and is mainly controlled by the mitochondrial genome. CMS is often found to be caused by chimeric mitochondrial open reading frames (ORFs) that are produced as a result of mitochondrial genome rearrangements.^11–14^ In many cases of CMS, male fertility can be restored by the introduction of nuclear genes known as restorer-of fertility (*Rf*) genes.

Although plant breeders have used CMS technology for producing F_1_ hybrids for enhancing crop productivity in numerous cereal and vegetable crops, the development of F_1_ hybrids has not been possible in legumes because of their high levels of self-pollination. In pigeonpea, however, a moderate level of insect-mediated out-crossing exists that could be used to develop a stable CMS system.^15^ In 2005, Saxena *et al.*^15^ derived a stable CMS system, ICPA 2039, from an interspecific hybrid of cultivated pigeonpea (*Cajanus cajan*) and a wild relative (*Cajanus cajanifolius*) (Supplementary Fig. S1). Previous CMS systems have been attempted in pigeonpea,^16,17^ but have been unsuccessful, mostly as a result of instability in the expression of male-sterility and -fertility restoration.^15^ The development and utilization of stable male-sterile lines from different cytoplasmic backgrounds are a key factor to the diversification of pigeonpea hybrid parental lines. Indeed, male-sterility systems in many crops do not allow the generation of completely male-sterile progenies, drastically limiting the use of male-sterile lines in F_1_ hybrid seed production.^18^ To accelerate hybrid pigeonpea breeding for yield and quality, understanding the molecular basis of male sterility is critically important. Specifically, the identification of CMS-associated genetic polymorphisms is a key pre-requisite for rational development of new and improved CMS systems for the production of superior F_1_ hybrids. Next-generation sequencing (NGS) has provided oppurtinities to gain the genetic information in a much faster and cost effective manner. NGS of mitochondrial genomes and analysis of genetic variations across the genomes of male-sterile, maintainer, and wild relative species will facilitate the identification of genetic features related to male sterility.

This study reports the generation and analysis of mitochondrial genome sequences of four *Cajanus* genotypes: the male-sterile line ICPA 2039, the maintainer line ICPB 2039, the hybrid line ICPH 2433, and the wild relative ICPW 29 (*C. cajanifolius*). A high-quality pigeonpea mitochondrial genome assembly has been developed for ICPA 2039. This study provides the first comparative study of legume mitochondrial genome sequences and identifies several re-arrangements and no-coverage regions (large regions >1000 bp; with zero coverage), as well as chimeric ORFs associated with CMS in pigeonpea.

## 2. Materials and methods

### 2.1. Plant material and mitochondrial DNA isolation

*Cajanus* lines ICPA 2039, ICPB 2039, ICPH 2433, and ICPW 29 were used as the source of mitochondrial DNA (mtDNA). mtDNA was isolated from 2-week-old etiolated seedlings and was purified before sequencing.^19^

### 2.2. Sequencing and assembly

**Figure 1.**
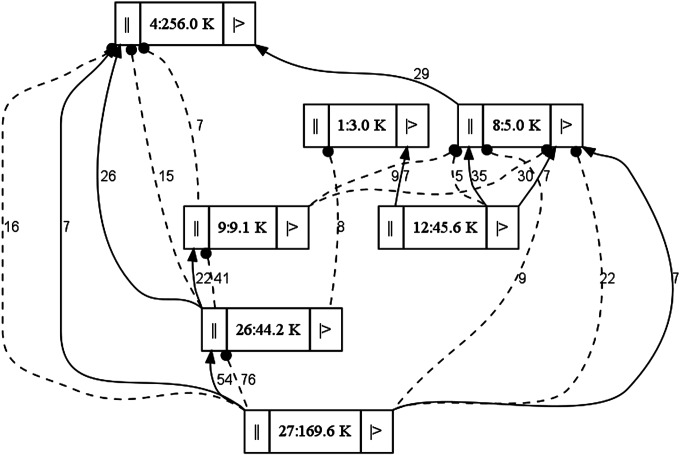
A scheme showing linking scaffold with the help of graph in a preliminary view of the assembly. Assembly graphs were used as a guide to connect the scaffolds. Each box represent a scaffold, ‘||’ represent the 3′ end, and ‘|>’ represent the 5′ end of each scaffold. Number on each scaffold represents the scaffold number and size of the scaffold. The thick black lines indicate that the scaffolds are attached in correct orientation and spotted line indicate that the scaffolds are attached in reverse orientation in the assembly. Numbers on these lines represents the sequence coverage. The orientation of each scaffold was confirmed through Sanger sequencing.

Mitochondrial genomes of four pigeonpea lines were pyrosequenced with the Roche/454 FLX sequencing platform following whole-genome amplification (WGA). WGA kit GenomePlex from Sigma (Sigma-aldrich, St. Louis, USA) was used in this study. Twenty nanograms of DNA template were used for WGA according to the protocol from manufactures. In summary, the WGA process was divided into fragmentation, library generation, and PCR amplification. The first two steps, fragmentation and library generation (3 kb of insert size), were carried out without interruption, to avoid the DNA degradation. Further to amplify higher amount of DNA, the GenomePlex reaction was allowed to proceed for 4 h. *De novo* genome assembly of the reference genome (ICPA 2039) was performed using Newbler, Celera, and CLC bio software programs. All the usable reads were aligned onto the contig sequences, and aligned paired-end sequences (PEs) were obtained. We then calculated the amount of shared PE relationships between each pair of contigs, weighted the rates of consistent and conflicting PEs, and then constructed the scaffolds step-by-step, beginning with the shortest insert-sized PEs, to long insert-sized PEs. Assemblies generated by the Newbler assembler were considered most robust in terms of length of the scaffolds and genome coverage and were used for further analysis. Gaps within the assembly were identified using contig-graph information. The Perl script, parse_link.pl, was used to identify and close the gaps *in silico*^20^ (http://www.cbcb.umd.edu/finishing/finishing-v1.tar.gz). Remaining gaps were filled by Sanger sequencing. Graphs were generated for a preliminary view of the assembly (Fig. 1) in an effort to check the order and orientation of mitochondrial scaffolds in the genome. Scaffolds that were not connected to other scaffolds in graph and showed low coverage were suspected to be part of the choloroplast genome. BLASTN searches were performed for these scaffolds against the NCBI database to validate these scaffolds are contamination from the chloroplast genome. Assembly graphs were used as a guide to connect the scaffolds. Primers were designed from the ends of the scaffolds that showed connections with other scaffolds in assembly graphs. The orientation of each scaffold within the assembly was confirmed by Sanger sequencing.

### 2.3. Gene prediction and annotation

Protein-coding and RNA genes were predicted by performing BLASTX and BLASTN searches, respectively, against a database of protein-coding, tRNA, and rRNA genes complied from all previously sequenced seed plant mitochondrial genomes.^21^ tRNAscan-SE^22^ was used to corroborate the tRNA boundaries identified by BLASTN. A BLAST score of *e*-value <1*e*−3 and percent identity threshold of >70% were initially used for filtering BLAST outputs. Gene boundaries were extended or trimmed to the positions of the start and stop codons manually using Artemis 12.0.^23^ Annotation data were written to a Sequin-formatted table file with a set of Perl and CGI scripts.

### 2.4. Gene-order comparison

To identify colinearity between the pigeonpea mitochondrial genome and other angiosperms, we used BLAT (Standalone BLAT v. 34) with an identity cut-off of ≥0.9 and coverage of ≥0.5 to compare the gene order of our *Cajanus* genomes in a pairwise fashion with that of the following 11 angiosperms: *Vigna radiata*,^24^ *Triticum aestivum*,^25^ *Oryza sativa*,^26^ *Zea mays*,^27^ *Arabidopsis thaliana*,^28^ *Beta vulgaris*,^29^ *Citrullus lanatus*,^21^ *Cucurbita pepo*,^21^ *Nicotina tabacum*,^30^ *Vitis vinifera*,^31^ and *Cucumis sativus*.^32^

### 2.5. Genome alignment and representation

The scaffolds of the three other mitochondrial genomes (ICPB 2039, ICPH 2433, and ICPW 29) were aligned with the finished assembly of ICPA 2039 genome using BLASTN. Circular genome representations of ICPA 2039 genome were generated using OGDRAW.^33^ Three different maps were generated to represent the alignment of ICPA 2039 genome with the three other genomes. Figures were scaled down to integrate all the four maps in a single map.

### 2.6. Identification of rearrangements and no-coverage regions

Comparative assemblies of all three genomes were generated using GS Reference Mapper 2.5. Raw reads of each mt genome were aligned to that of ICPA 2039 genome assembly in order to detect any sequence-level differences between them. Rearrangements with >60% frequency were considered for further analysis. No-coverage regions were extracted using a custom Perl script, which checks the coverage of every base in the assembly and groups the consecutive positions where coverage is very low. Regions >1 kb and with approximately zero coverage were considered as no-coverage regions.

### 2.7. Chimeric ORFs

Sequences for ORFs >100 codons in the vicinity of rearrangements or within no-coverage regions were collected using Artemis 12.0.^23^ ORFs coding for known mitochondrial genes were excluded from the analysis. Further, these ORFs were blasted against the ICPA 2039 genome itself in order to check whether these ORFs carry part of other genes or ORFs. First hit of the blast match were left as that will be the original location of these ORFs. All the other hits showing identity ≥95% and sequence coverage of ≥16 bp were considered. Further, these ORFs were checked in terms of their closeness to any predicted gene. Potential transmembrane helices were predicted with TMHMM 2.0.^34^ A scoring crietria from 0 to 4 was assigned to each ORF, one for the presence of parts of other genes, one for the proximity of any predicted genes, one for the presence of hydrophobic domains, and one additional score for carrying parts of *atp* genes. ORFs showing score of ≥3 were considered as the potential chimeric ORFs.

## 3. Results and discussion

Pigeonpea is an important legume crop for resource-poor smallholder farmers in marginal environments. Unfortunately, the productivity of this legume staple crop has stagnated at ca. 750 kg/ha due to its exposure to biotic and abiotic stresses. Pigeonpea recently became the first legume to have F_1_ hybrids released based on a CMS system.^35^ The initial pigeonpea F_1_ hybrids (e.g. ICPH 2671) showed >30% yield advantages over the best pure line varieties in the same geographic regions. Such advances clearly indicate that pigeonpea F_1_ hybrid technology has the potential to break the current yield plateau. For successful and sustainable pigeonpea hybrid production and extension, the following are critical factors: (i) diversification of parental lines and CMS sources, (ii) improvement of parental lines for tolerance to biotic and abiotic stresses, and (iii) maintaining the purity of hybrid seeds. In this context, improvement of parental lines is underway through conventional and molecular breeding approaches that are being accelerated by the availability of a sequenced pigeonpea genome.^36^ Simple sequence repeat (SSR) markers-based F_1_ hybrid purity testing has also been initiated for ensuring the purity of hybrid seeds.^37,38^ However, major challenges remain in relation to the need for diversification of CMS sources in the pigeonpea genepool. Although seven cytoplasmic sources are available (*Cajanus sericeus*, *C. scarabaeoides*, *C. volubilis*, *C. cajanifolius*, *C. cajan*, *C. lineatus*, and *C. platycarpus*), only *C. cajanifoius* has currently been commercially exploited. The other six sources have not been able to be used commercially, because they express the CMS trait at an adequate level. To understand the factors conditioning the efficiency differences between sources of CMS, it will first be necessary to understand the molecular basis of CMS in pigeonpea.

### 3.1. Sequencing and assembly of four mitochondrial genomes of pigeonpea

We used Roche/454 FLX technology, targeted Sanger sequencing, and computational approaches to produce one complete and three draft assemblies for the mitochondrial genomes of four *Cajanus* lines. Northern blot-based screening and shotgun sequencing have been the conventional approaches used to identify CMS-associated chimeric ORFs in different plant species, including maize,^39^ sugar beet,^40^ rice,^41,42^ wheat,^25^ and brassica.^43^ Recently, Bentolila and Stefanov^44^ successfully identified a candidate for the wild abortive CMS-encoding gene by pyrosequencing of two rice mitochondrial genomes using Roche/454 sequencing technology. However, Northern screening approaches used for the same rice genomes failed to identify any potential CMS candidates in wild abortive rice CMS lines.^44^ Therefore, we used Roche/454 sequencing technology and a *de novo* assembly approach to sequence the mitochondrial genome of *Cajanus* species. Due to the unknown genome architecture of the pigeonpea mitochondrial genome, we did not rely entirely on *in silico* approaches, but also validated proposed interscaffold connections by Sanger sequencing. To identify regions which varied in a manner correlated with CMS, the four mitochondrial genomes were then compared using the ICPA 2039 assembly as a reference.

Roche/454 sequencing of four genomes from purified mtDNA generated totals of 38.8, 15.6, 37.1, and 23.8 Mb of paired-end data for ICPA 2039, ICPB 2039, ICPH 2433, and ICPW 29, respectively. The sequencing reads were assembled into scaffolds using three different *de novo* assembly programs—Newbler, CLCBio, and Celera. Assemblies generated by the Newbler assembler were considered as the best assemblies (Table 1), with scaffold N50 values of 169.6, 1.2, 169.9, and 108.1 kb for ICPA 2039, ICPB 2039, ICPH 2433, and ICPW 29, respectively. Additional sequence data were generated for the comparatively under-sequenced genotypes of ICPB 2039 (15.1 Mb) and ICPW 29 (23.2 Mb). The sequencing data for these were reassembled and N50 of two assemblies thus improved from 1.2 to 12.8 kb for ICPB 2039 and from 108.2 to 159.2 kb for ICPW 29. In summary, mean scaffold lengths of 22.4, 8.8, 6.3 and 37.3 kb were achieved for the pigeonpea mitochondrial genomes of lines ICPA 2039, ICPB 2039, ICPH 2433, and ICPW 29, respectively. Analysis of sequence data for GC content indicated similar GC content distribution in all of the four genomes (Supplementary Fig. S2).

**Table 1.** Generation of 454/FLX data and assembly statistics of ICPW 29, ICPA 2039, ICPB 2039, and ICPH 2433

Genotypes	Number of sequence reads (length) generated	Newbler					Celera				CLC Bio	
		Number of scaffolds	Bases in scaffolds (bp)	N50 scaffold size (bp)	Number of large contigs^a^	Bases in large contigs (bp)	Number of scaffolds	Bases in scaffolds (bp)	Number of big contigs^b^	Big contig length (bp)	Number of contigs	Bases in contigs (bp)
ICPW 29	74 109 (23.8 Mb)/164 071 (53.4 Mb)^c^	156/18^c^ (4)^d^	723 814/672 137^c^ (575 487)^e^	108 193/159 243^c^	202/425^c^	694 265/858 696^c^	34	618 692	15	501 689	392	951 760
ICPA 2039	121 170 (38.8 Mb)	30 (7)^d^	672 918 (532 372)^e^	169 595	345	828 279	84	662 357	20	475 091	1348	1 782 550
ICPB 2039	51 723 (15.6 Mb)/117 163 (36.4 Mb)^c^	387/52^c^ (34)^d^	415 181/459 802^c^ (335 926)^e^	1153/12 823^c^	430/669^c^	404 882/716 244^c^	113	199 602	0	0	1032	529 436
ICPH 2433	116 021 (37.1 Mb)	108 (9)^d^	681 810 (539 865)^e^	169 903	184	677 158	44	577 054	18	468 611	564	1 227 305

^a^Newbler assembler classifies contigs >500 bp as large contigs.

^b^Celera assembler classifies contigs >10 kb as big contigs.

^c^Data and assembly statistics of ICPW 29 and ICPB 2039 additional reads.

^d^Number of scaffolds from the mitochondrial genome.

^e^Base in scaffolds from the mitochondrial genome.

### 3.2. Finishing of reference mitochondrial genome ICPA 2039

The scaffolds of ICPA 2039 were further refined by removing contamination from nuclear or chloroplast genomes, and by closing gap regions within these scaffolds using Sanger sequencing. A preliminary view of the ICPA 2039 assembly was generated to check the connections between the scaffolds and for the removal of contaminants (Fig. 1). Scaffolds from the mitochondrial genome assembly were selected based on their coverage and links with the other scaffolds as shown in Fig. 1. Selected scaffolds were subjected to BLASTN analysis against the NCBI database. Of 30 selected scaffolds of ICPA 2039, with a total length of 672 918 bp, seven were confirmed to be mitochondrial in origin, representing 532 372 bp or 79% of the total sequence data (Table 1). Seven scaffolds were homologous to cpDNA, representing 78 461 bp (11.6% of the total sequence). Eleven further scaffolds matched nuclear DNA representing 46 228 bp (6.9% of the sequence). Two scaffolds representing 8268 bp (1.2% of the sequence data) matched the sequence of the plasmid DNA. The remaining three scaffolds representing 8309 bp (1.2%) of sequence data did not show any match in the NCBI database and may represent sequences unique to pigeonpea. The seven scaffolds that matched other plant mtDNA were targeted for further analysis. A total of 38 gaps (26 830 bp) were observed in the seven mitochondrial scaffolds, represented by Ns in the assemblies. Using the parse_link.pl script (http://www.cbcb.umd.edu/finishing/finishing-v1.tar.gz) from the finishing toolbox (see Materials and Methods), 47 contigs from contigs that had not previously been assembled into scaffolds were introduced to fill 38 gaps inside the seven scaffolds. Two gaps (of 65 bp each), which the script was unable to fill *in silico*, were closed using Sanger sequencing technology. Subsequently, assembly graphs were used as a guide to connect the scaffolds. To confirm the order and orientation of each scaffold within the assembly, primer pairs were designed from the ends of each scaffold based on their connections with other scaffolds in assembly graphs. A set of 24 primer pairs (Supplementary Table S1) were used to generate amplicons and sequence data were generated for these using Sanger sequencing technology. In this way, a high-quality, circular-mapping mitochondrial genome of 545 742 bp in total length, with ∼23-fold coverage, was assembled for ICPA 2039. This master circular molecule contains a large recombinationally active repeat of size 4951 bp, which is extending from positions 531 745 to 536 696 bp. Recombinationally active large repeats are a very common feature of plant mitochondrial genomes.^45,46^

### 3.3. Gene content

Within the mitochondrial genome of ICPA 2039, we identified 34 protein-coding, 14 tRNA, and 3 rRNA genes (Supplementary Table S2), for a total of 29 346 bp of protein exons; 31 018 bp of intronic sequence; 5255 bp of rRNA genes; and 1477 bp of tRNA genes (Table 2). We did not find a copy of, cox2, confirmed proposals that this gene has lost in the legume lineages.^24,47^ In contrast, we found ICPA 2039 to contain two identical copies of the cox3 gene. Multiple copies of tRNAs for cysteine, lysine, and methionine were observed to be present in the mitochondrial genome of ICPA 2039. The tRNA genes, carrying methionine, are also highly similar to those of the plastid as is likely derived from the cpDNA, as is that of tryptophan (Supplementary Table S2).

**Table 2.** Genome coverage by coding features in ICPA 2039 mitochondrial genome assembly

Class	Feature	ICPA 2039^a^ (%)
Total size		545 742 bp
Coding	Protein exons	29 346 bp (5.4)
	Introns	31 018 bp (5.6)
	rRNA	5 255 bp (0.9)
	tRNA	1477 bp (0.2)
Non-coding	Mitochondria-like	220 747 bp (40.5)
	Nuclear-like	40 330 bp (7.4)

^a^Figure in parentheses represents the percentage of total size.

To annotate mtDNA-encoded genes in the other sequenced lines, their scaffolds were compared with ICPA 2039. Six scaffolds derived from the ICPH 2433 hybrid contained 32 protein-coding genes and 12 tRNA genes between them (Supplementary Table S3). Four mitochondrial scaffolds of ICPW 29 covered 33 protein-coding and 14 tRNA genes (Supplementary Table S4). As in ICPA 2039, multiple copies of cysteine, lysine, and methionine-tRNA genes were present in the scaffolds of ICPH 2433 and ICPW 29. Due to low sequence coverage and small scaffold size, the 17 mitochondrial scaffolds of ICPB 2039 line only covered 15 protein and 11 tRNA genes between them (Supplementary Table S5).

### 3.4. Structural features of the pigeonpea mitochondrial genome compared with other plant species

The assembled *Cajanus* mitochondrial genome was compared with the mitochondrial genomes of 11 other land plant species. The species for comparison include one legume—*V. radiata*,^24^ three cereals—*T. aestivum*,^25^ *O. sativa*,^26^ *Z. mays*,^27^ and seven other eudicots, including *A. thaliana*,^28^ *B. vulgaris*,^29^ *Citrullus lanatus*,^21^ *Cucurbita pepo*,^21^ *N. tabacum*,^30^ *Vitis vinifera*,^31^ and *Cucumis sativus*.^32^ In terms of mitochondrial genome size, the pigeonpea mitochondrial genome (545 742 bp) is substantially larger than the mitochondrial genome of closest sequenced legume species *V. radiata*^24^ (401 262 bp). The size of pigeonpea mitochondrial genome was found to be comparable in size with the mitochondrial genomes of cereal species, e.g. *O. sativa* (490 kb), *Z. mays* (569 kb), and greater than the median angiosperm mitochondrial genome size (473 kb). Mitochondrial genome size can not only vary between different species, but can also show variations between different lines of the same species. For instance, the genome size of five sequenced maize mitochondrial genomes is known to vary from 535 825 to 739 719 bp.^39^ The plant mitochondrial genomes are rich in non-coding regions and are highly variable in their non-coding regions. In our analysis, 12.29% of the pigeonpea mitochondrial genome was covered by the coding regions, which is lower than but comparable to *V. radiata*^24^ (16.88%), *Brassica napus*^43^ (17.34%), and *Citrullus lanatus*^21^ (18.8%). However, the coding regions of the pigeonpea mitochondrial genome were found to be greater than the *Cucurbita pepo* in which only 6.9%^21^ of the mitochondrial genome is covered by coding regions. *Cucurbita pepo* has a particularly large mitochondrial genome (982 833 bp), due to the insertions of chloroplast (>113 kb) and short repeated sequences (>370 kb) in the mitochondrial sequences.^21^ The available plant mitochondrial genome sequences therefore suggest that gene composition does not depend on total genome size. Gene number can be conserved despite changes in size derived from the insertion of repetitive sequences, or of sequences transferred from the chloroplast and nucleus into intergenic regions. In addition, a large number of rearrangements of genes and ORFs are common features in plant mitochondrial genomes.

### 3.5. Comparison of gene order with other plant species

**Figure 2.**
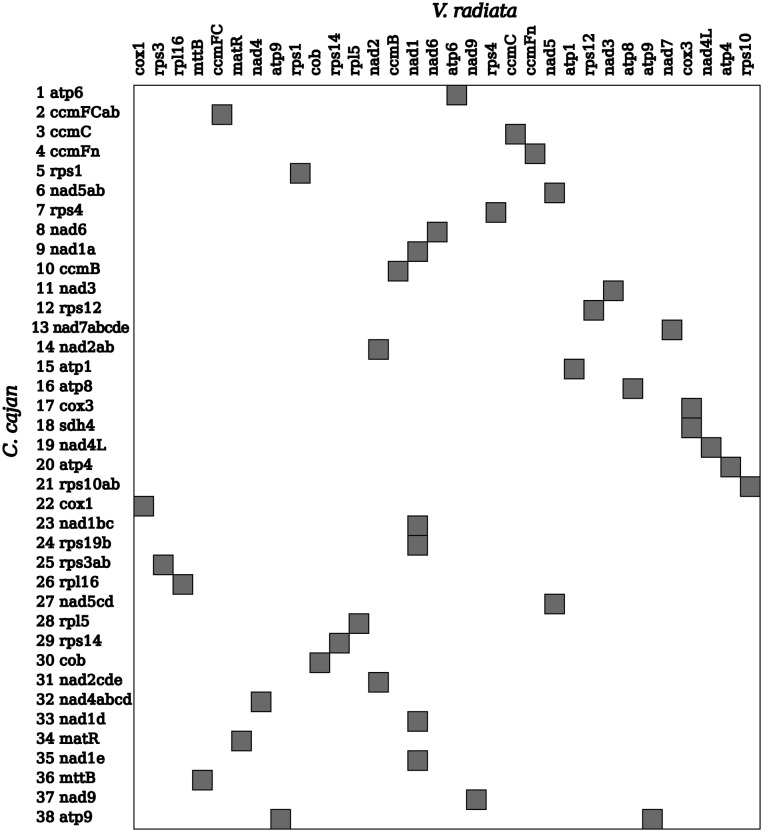
Correlation of gene order between the mitochondrial gene maps of *C. cajan* and *V. radiata*. Left-hand side is represented by genes identified in *C. cajan* and top side is represented by genes of *V. radiata*. Shaded blocks in the image represent the correlation of gene orders.

We also compared gene maps of protein-coding and rRNA genes encoded by the pigeonpea mitochondrial genome (ICPA 2039) with those of the other 11 sequenced plant species. Unsurprisingly, the highest level of synteny for the mitochondrial genomes was observed between *C. cajan* and the related legume *V. radiata*. For instance, one four-gene cluster (*cox3*-*nad4L*-*atp4*-*rps10ab*), two three-gene clusters (*nad6*-*nad1*-*ccmB* and *rpl5*-*rps14*-*cob*), and three two-gene clusters (*rps3ab*-*rpl16*, *rps12*-*nad3*, and *ccmC*-*ccmFn*) were syntenic between these two species (Fig. 2). On the other hand, only two two-gene clusters (*nad3*-*rps12* and *ccmC*-*ccmFn*) showed synteny in mitochondrial genomes of *C. cajan* with three cereals species analysed (Supplementary Figs S3–S5). Two two-gene clusters (*nad2ab*-*atp1* and *rps3ab*-*rpl16*) of the *C. cajan* mitochondrial genome showed synteny when compared with wheat and maize (Supplementary Figs S3 and S4). The most highly conserved gene cluster was the two-gene cluster of *rps3ab*-*rpl16*, which was syntenic across 7 of the 11 species (Fig. 2 and Supplementary Figs S3–S12).

### 3.6. Comparison of mitochondrial genome sequences among *Cajanus* lines

**Figure 3.**
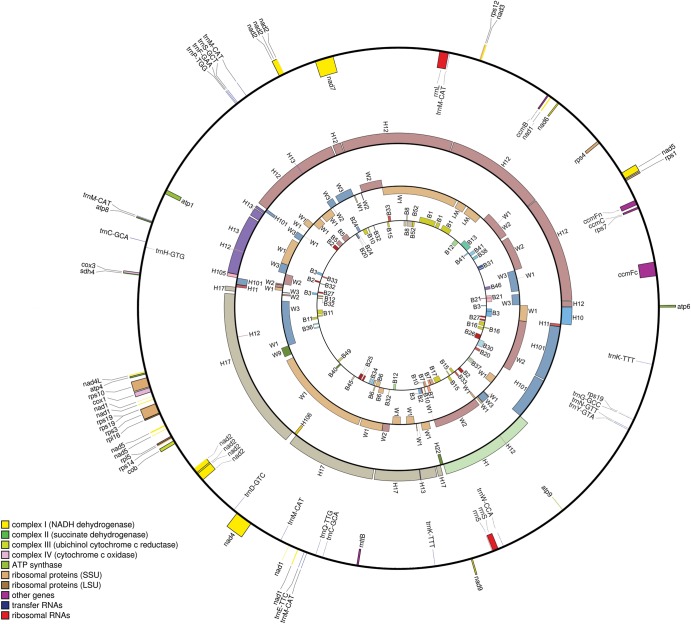
Alignments of *Cajanus* mitochondrial genomes. The outer circle represents the finalized mitochondrial genome assembly and gene annotation of male-sterile line ICPA 2039. Second, third, and fourth circles from the outer circle represent the scaffolds of ICPH 2433, ICPW 29, and ICPB 2039 mapped on ICPA 2039 assembly. Numbers on each circle represent the scaffolds of each line. Hn represent the scaffolds for ICPH 2433, W*n* represent the scaffolds for ICPW 29 and B*n* represent the scaffolds for ICPB 2039, where *n* is the scaffold number.

Comparisons of gene order typically highlight the high rate of mitochondrial genome rearrangements between different plant species. Our analysis indicates that the mitochondrial genome of pigeonpea shares only six gene clusters with the closely related sequenced species *V. radiata*,^24^ which between them cover only 16 genes. Fewer gene clusters was observed to be conserved in comparison with more distantly related species. Due to the dynamic nature of the plant mitochondrial genomes, extensive structural variations can also be expected to occur in different lines of a given species.^46^ Comparison of mitochondrial genomes from five different lines of maize revealed 16 rearrangements, even between two fertile cytotypes.^39^ To understand the patterns of genetic variation associated with CMS in pigeonpea, and its effects on maternal inheritance, we first aligned the scaffolds of the mitochondrial genomes of the three *Cajanus* lines (i.e. ICPB 2039, ICPH 2433, and ICPW 29) using BLASTN, together, with that of the male-sterile line ICPA 2039. This demonstrated that the genomes of ICPA 2039 and ICPB 2039 are highly diverged from each other. Conversely, the mitochondrial genome of the hybrid ICPH 2433 (produced from the ICPA 2039 × ICPR 2433 cross) showed the highest level of synteny with the ICPA 2039 line, followed by ICPW 29 (Fig. 3), supporting the model that the CMS trait is maternally inherited in pigeonpea hybrids.

The sequence-level divergence between the mitochondrial genomes of ICPA 2039 and ICPB 2039 lines identified by BLASTN was further validated by use of the GS Reference Mapper 2.5, which is commonly used for mapping 454 reads to a reference assembly. Mapping raw reads of ICPB 2039, ICPH 2433, and ICPW 29 on to the assembly of ICPA 2039, a maximum number of rearrangements were observed in the fertile and sterile lines of ICPA 2039 system (Supplementary Fig. S1). While building the comparative assemblies, we identified the no-coverage regions along with the rearrangements in order to reduce the effects of sequencing artifacts in further comparison. Twenty-two rearrangements and 17 no-coverage regions were observed in ICPB 2039 compared with ICPA 2039. Using the same criteria, 9 rearrangements and 12 no-coverage regions were observed in the mitochondrial genome of ICPW 29 when compared with that of ICPA 2039. These nine rearrangements could be the result of differences occurred during the maintenance of the CMS cytoplasm. We do not expect these differences to be associated with the CMS trait as the wild relative line is the maternal parent of the sterile line. The mitochondrial genome of ICPH 2433 was found to be closest in sequence to that of ICPA 2039, with no differences observed between these lines (Supplementary Fig. S13 and Supplementary Tables S6 and S7).

### 3.7. Candidate CMS-associated chimeric ORFs

The CMS trait is often associated with chimeric ORFs that are the products of mitochondrial genome rearrangement,^48^ which can cause pollen abortion. In some crops, chimeric ORFs are found in sterile lines (e.g. A-line), but absent in fertile lines (e.g. B- and R-lines).^13,14,39^ A number of studies have confirmed the role of chimeric ORFs in male sterility by disrupting the function of ORFs by inserting or deleting a few base pairs (reviewed by Hanson and Bentolila).^48^ Many of the chimeric genes associated with CMS in other crops are found in the proximity of protein-coding genes and include regions encoding transmembrane domains and other parts of known mitochondrial genes.^48–52^ Hence, we set out to identify those chimeric ORFs, which most closely resemble these criteria by scanning around positions that have undergone rearrangements or which are absent from particular pigeonpea lines. Only ORFs >300 bp were considered, and were ranked as chimeric based on the presence of parts of other genes, proximity to known mitochondrial genes, and the presence of hydrophobic domains. A scoring systems ranging from 0 to 4 was assigned to each ORF (see Chimeric ORFs section of Materials and methods). As abnormal *atp* synthase genes are sometimes associated with CMS,^49–52^ ORFs containing parts of *atp* genes were more heavily weighted.

Our study identifies 13 such potential CMS candidates in the pigeonpea male-sterile line ICPA 2039 (Table 3). Of these 13 potential candidates, five carry parts of other mitochondrial genes and eight were observed to be in the proximity of other mitochondrial genes. Liu *et al.*^25^ have hypothesized that a wheat K-type CMS line, Ks3, contains a chimeric ORF encoding partial subunits of several components of the respiratory chain complex, including, *atp4*, *atp6*, *nad3*, *nad6*, *nad9*, *cox1*, and *cox3*. These altered proteins may interfere with the normal function of respiratory chain reactions and cause pollen development to abort. Intriguingly, five of the candidates identified in our study incorporate parts of some of these genes including, *atp1*, *nad4*, *rps4*, *nad5*, and *atp9*. This presents the possibility of a similar mechanism in pigeonpea CMS as found in rice. Transmembrane domains are another prominent feature associated with CMS ORFs.^53^ Many of our candidate ORFs carry regions predicted to encode transmembrane domains, and a number of the encoded proteins have been shown to be associated with the inner mitochondrial membrane.^48^ Recently, Bentolila and Stefanov^44^ have identified a candidate for the wild abortive CMS in rice that has arisen via rearrangement, is chimeric in structure, possesses predicted transmembrane domains, as well as possess the promoter of a mitochondrial gene. Of our 13 candidates, 11 are predicted to carry such transmembrane domains. These novel ORFs may trigger CMS by damaging mitochondrial membrane structure such that the resulting permeability change affects mitochondrial function.^48,54^ Previous histological studies of CMS in pigeonpea have revealed that meiosis in both male-fertile and male-sterile plants proceeds normally up to the tetrad stage, and that during this period, the tapetum remains intact. Male sterility becomes manifest after this, with tetrads in male-sterile plants remaining enclosed within a persistent tetrad wall and subsequently undergoing vacuolation and abortion of pollen grains.^55^ Therefore, identifying the ORFs that are causative for CMS in pigeonpea will require the transcription and translation patterns of our unique ORF candidates to be determined, including in young to mature buds, floral parts including the pollen mother cell, tetrad, and pollen grains. The roles of transmembrane domains and respiration in the mitochondrial genome of ICPA 2039 will also need to be assessed. Future, structural and functional studies will allow the exact mitochondrial genomic segments responsible for male sterility in pigeonpea to be defined.

**Table 3.** Potential chimeric ORFs identified from the no-coverage and rearrangement regions between the ICPA 2039 and ICPB 2039 lines

ORF start	ORF stop	ORF length	Nearest gene	Subject start	Subject stop	Chimera length	Identity	Subject features	No of transmembrane helices
260 331	260 702	371	*cox3*	260 342	260 702	361	98	ORF	1
164 867	165 424	557	*nad7*	165 424	165 353	72	100	ORF	1
420 342	420 902	560	—	233 322	232 862	461	96	*atp1*	0
534 744	535 115	371	—	352 424	352 403	361	98	*nad4*	1
264 435	265 745	1310	—	60 093	59 813	281	100	*rps4*	2
165 464	165 853	389	*nad7*	265 742	265 981	241	97	ORF	3
164 867	165 424	557	*nad7*	165 424	165 353	72	100	ORF	1
276 037	276 405	368	—	468 809	468 752	58	98	*atp9*	3
396 025	396 876	851	*mttB*	264 909	265 396	488	97	ORF	2
396 285	396 641	356	*mttB*	265 144	265 396	253	99	ORF	1
264 069	264 434	365	—	44 935	45 092	158	100	*nad5*	1
165 633	166 088	455	*nad7*	265 981	265 886	96	100	ORF	0
8534	8842	308	*ccmFc*	476 033	476 052	20	100	—	1

## Accession numbers

Genome sequences and annotations from this article have been submitted to the GenBank data library under accession number SRA053693.

## Authors' contribution

R.T. and R.K.S. performed experiments, analysed data and contributed to writing the manuscript; J.D., T.S., W.C., G.F., A.J.A., and C.S. contributed to analysis data and interpretation of result; Y.L.X. and C.D.T. performed experiments and analysed data, and R.K.V. conceived and guided experiments, contributed to analysis of data, interpreted results, contributed to writing and the finalized manuscript.

## Supplementary data

Supplementary data are available at www.dnaresearch.oxfordjournals.org.

Supplementary Data
