# Supplementary material for: Cytoplasmic Male Sterility-Associated Chimeric Open Reading Frames Identified by Mitochondrial Genome Sequencing of Four Cajanus Genotypes
Source: DNA Res. 2013 Jun 20;20(5):485–95. doi: 10.1093/dnares/dst025 (PMC3789559; doi:10.1093/dnares/dst025)
Supplement: Supplementary Data [file supp_20_5_485__index.html]

Cytoplasmic Male Sterility-Associated Chimeric Open Reading Frames Identified by Mitochondrial Genome Sequencing of Four Cajanus Genotypes — Supplementary Data 

# Cytoplasmic Male Sterility-Associated Chimeric Open Reading Frames Identified by Mitochondrial Genome Sequencing of Four *Cajanus* Genotypes

## 

Supplementary Data

**Files in this Data Supplement:**

- Supplementary Data - Pdf file
