# Supplementary material for: Cytoplasmic Male Sterility-Associated Chimeric Open Reading Frames Identified by Mitochondrial Genome Sequencing of Four Cajanus Genotypes
Source: DNA Res. 2013 Jun 20;20(5):485–95. doi: 10.1093/dnares/dst025 (PMC3789559; doi:10.1093/dnares/dst025)
Supplement: Supplementary Data [file supp_dst025_dst025supp.pdf]

Table S1: Details on 24 primer pairs designed from the end-sequences of the scaffolds to confirm the orientation in the mitochondrial genome assembly of ICPA 2039.

Primer name	Forward primer (5'-3')	Reverse primer (5'-3')	Tm (°C)	Product size (bp)
*CcMtM01	AGGAGACAGCTGATGGTGCT	CCTTGGGGGATACACCTTCT	60.017	132
CcMtM02	CCCAGCTAAAGCGAATCTCA	ACTGGTGCTTACACCGAAGC	60.481	106
CcMtM03	TATCTTGCTTGAGAGGGGGA	GAACTCAAAGAGCGAGCCAC	59.767	215
CcMtM04	TAGCGAAGGAGAGGGTGAGA	GCAATGATTTCAGACGCAGA	60.088	257
CcMtM05	GGAGGTTGAGGTCTGTGTCC	TGAGGCTAGGCCTTATTGGA	59.534	181
CcMtM06	AGGCCCAACAACCTAAAAGG	AAGGTACGGAGTCGCTCAAC	60.345	228
CcMtM07	CTCGAGTTTCTTGGTCGGTC	TTTCTCAGGGGATGAGGTTG	59.844	153
CcMtM08	TAAAGCAGTGTGCGTCATCC	CGCCCACCTCATTCATATCT	59.871	115
CcMtM09	GAGCATAAGGATTAGCAGAATCG	CAAGTGCTACGAGTGCTTCG	59.411	104
CcMtM10	TGGGTCCAAAGGACAGGTAG	AGAAAATCTGCCTCCAGCAA	59.959	139
CcMtM11	CCAACCTCTCGAAATCCAAA	TCAGATGATTTGTGGACGGA	60.044	107
CcMtM12	ACAGACCAAGCAAGGGCTTA	AGGGGCTTAAAGGAGTTTCG	59.875	147
CcMtM13	CTGAGAGAAGGCCTGTGACC	ATTATTTCCACCCCCTCGTC	59.986	273
CcMtM14	ACCCACAATCAGCCAAGTTC	CGAGGTCTCAACGAAAGGAG	59.973	185
CcMtM15	ACTCCGTGCAGGAAAGACTC	GAGCGGCCTGTAATAAGTGC	59.455	238
CcMtM16	CGGGGTTTGATAGTTGCAGT	CAATCCCTCTTTCTGAAGCG	59.993	111
CcMtM17	AAAAGCGTGTTCCCTTCAGA	TAAAGGAAAGGCTCGACGAA	59.853	142
CcMtM18	TTCCACTTGCTTTCTCGCTT	AGTTGGTGAAACCTGAGCGT	60.132	187
CcMtM19	CCTCTTCCCAGGAATGAACA	AAGCAAATAGATAGCCCCCG	60.042	168
CcMtM20	TGCTCTCTTGCTACGGGATT	ACGCTGGTCCATCACTTACC	59.978	203
CcMtM21	CCTCGTGGGGAAAGATAAGA	TATCTCCCCCTTGCCTTTTT	59.122	234
CcMtM22	CAGAACGAATCTCTTATCGCC	ACTCCAGTCCTACCCCGTCT	58.941	175
CcMtM23	GTTTCGGTACGAAAGCCTGA	TCCGTCTTGCCTTTGCTACT	60.249	202
CcMtM24	AGTCCCGGGATCGTAAAGTT	TGTCACAAGGAAGGTGGTCA	59.827	146

*CcMtM: *Cajanus cajan* mitochondria marker.

Table S2: Genes in pigeonpea mitochondrial genome (ICPA2039)

**Genes**	**Size**	**Coordinates**		**Strand**	**Organism with top hit**
		**From**	**To**		
I. Complex I genes					
nad1a	383	82256	81873	-	*Vigna radiata*
nad1b	88	302673	302761	+	*Vigna radiata*
nad1c	194	303623	303817	+	*Vigna radiata*
nad1d	86	371688	371774	+	*Vigna radiata*
nad1e	254	374878	375132	+	*Vigna radiata*
nad1	**1005**	-	-	**x**	-
nad2a	152	175842	175994	+	*Vigna radiata*
nad2b	401	177235	177636	+	*Vigna radiata*
nad2c	158	335490	335332	-	*Vigna radiata*
nad2d	638	332814	332176	-	*Vigna radiata*
nad2e	170	330741	330571	-	*Vigna radiata*
nad2	**1519**	-	-	**x**	-
nad3	353	108269	108622	+	*Vigna radiata*
nad4a	458	351289	351747	+	*Vigna radiata*
nad4b	584	353136	353720	+	*Vigna radiata*
nad4c	419	356763	357182	+	*Vigna radiata*
nad4d	86	359741	359827	+	*Vigna radiata*
nad4	**1547**	-	-	**+**	-
nad4L	296	294002	294271	+	*Vigna radiata*
nad5a	236	45291	45055	-	*Vigna radiata*
nad5b	1232	47332	46100	-	*Vigna radiata*
nad5c	395	313079	313474	+	*Vigna radiata*
nad5d	137	314387	314524	+	*Vigna radiata*
nad5	**2000**	-	-	**x**	-
nad6	614	79231	78617	-	*Vigna radiata*
nad7a	161	164750	164589	-	*Vigna radiata*
nad7b	101	163739	163638	-	*Cucurbita pepo*
nad7c	464	162302	161838	-	*Vigna radiata*
nad7d	245	160788	160543	-	*Vigna radiata*
nad7e	323	158734	158411	-	*Vigna radiata*
nad7	**1294**	-	-	**-**	-
nad9	569	433769	434338	+	*Vigna radiata*
II. Complex II genes					
sdh4	239	261724	261963	+	*Citrullus lanatus*
III. Complex III genes					
cob	1172	319048	320220	+	*Cycas taitungensis*
IV. Complex IV genes					

cox1	1579	299624	301203	+	*Vigna radiata*
cox3	794	260999	261793	+	*Vigna radiata*
V. Complex V genes					
atp1	1511	233337	231826	-	*Vigna radiata*
atp4	566	294470	295036	+	*Vigna radiata*
atp6	728	301	1029	+	*Carica papaya*
atp8	482	243540	244022	+	*Vigna radiata*
atp9	170	468828	468658	-	*Vigna radiata*
VI. Cytochrome c biogenesis genes					
ccmB	620	83549	82929	-	*Vigna radiata*
ccmC	740	34816	34076	-	*Vigna radiata*
ccmFCa	779	15633	14854	-	*Vigna radiata*
ccmFCb	566	11352	10786	-	*Vigna radiata*
ccmFC	**1345**	-	-	**-**	-
ccmFn	1733	37237	35504	-	*Vigna radiata*
VII. Ribosomal protein genes					
rpl16	510	308657	309167	+	*Arabidopsis thaliana*
rpl5	554	317272	317826	+	*Vigna radiata*
rps1	620	44252	44872	+	*Carica papaya*
rps10a	309	296174	296483	+	*Vigna radiata*
rps10b	107	299315	299422	+	*Vigna radiata*
rps10	**416**	-	-	**+**	-
rps12	374	108674	109048	+	*Vigna radiata*
rps14	299	317837	318136	+	*Vigna radiata*
rps19a	68	507787	507719	-	*Cucurbita pepo*
rps19b	119	305180	305299	+	*Cucurbita pepo*
rps19	**187**	-	-	**x**	-
rps3a	74	305316	305390	+	*Beta vulgaris*
rps3b	1571	307156	308727	+	*Beta vulgaris*
rps3	**1645**	-	-	**+**	-
rps4	1037	60663	59626	-	*Vigna radiata*
rps7	89	34089	34000	-	*Citrullus lanatus*
VIII. Other protein coding genes					
matR	2017	372328	374345	+	*Carica papaya*
mttB	722	395960	395238	-	*Nicotiana tabacum*
IX. rRNA genes					
rrn5	113	441424	441311	-	*Vigna radiata*
rrnL	3154	122673	119519	-	*Vigna radiata*
rrnS	1988	443580	441592	-	*Vigna radiata*
X. tRNA genes					

trnN	71	504005	503934	-	*Beta vulgaris*
trnD	73	349884	349811	-	*Nicotiana tabacum*
trnC-1	72	250520	250592	+	*Vigna radiata*
trnC-2	70	383649	383579	-	*Vigna radiata*
trnQ	71	381197	381126	-	*Cucumis sativus*
trnE	71	377238	377309	+	*Arabidopsis thaliana*
trnG	71	507338	507267	-	*Beta vulgaris*
trnH	73	252351	252278	-	*Cucumis sativus*
trnK-1	72	421642	421570	-	*Vigna radiata*
trnK-2	72	526879	526807	-	*Vigna radiata*
trnM-1	73	119146	119073	+	*Nicotiana tabacum*
trnM-2	73	190371	190444	+	*Nicotiana tabacum*
trnM-3	73	243291	243364	+	*Nicotiana tabacum*
trnM-4	81	368552	368471	-	*Zea mays*
trnM-5-cp	72	378147	378219	+	*Citrullus lanatus*
trnF	73	194491	194564	+	*Vigna radiata*
trnP	74	194824	194898	+	*Cucumis sativus*
trnS	87	194042	194129	+	*Zea perennis*
trnW-cp	73	445511	445438	-	*Vigna radiata*
trnY	82	503562	503480	-	*Vigna radiata*

a. Boldface, sum of all exons; lower-case letters, exons of a protein-coding gene; hyphenated, copies of the same gene; cp, probable chloroplast origin. b. Plus and minus, coded by the forward and reverse strand; x, trans-spliced gene.

Table S3: Gene annotation of scaffolds derived from mitochondrial sequences of ICPH 2433.

ScaffoldID	Genes	Size	Coordinates		Strand	Organism with top hit
			From	To		
Scaffold1	*atp9*	221	35152	34931	-	*Vigna radiata*
	nad9	569	94	663	+	*Vigna radiata*
Scaffold11	*cox3*	794	1284	490	-	*Vigna radiata*
	sdh4	239	559	320	-	*Citrullus lanatus*
Scaffold12	*atp6*	749	543	1292	+	*Carica papaya*
	ccmB	617	83861	83244	-	*Vigna radiata*
	ccmC	737	35131	34394	-	*Vigna radiata*
	ccmFCa	780	15949	15169	-	*Vigna radiata*
	ccmFCb	565	11667	11102	-	*Vigna radiata*
	ccmFn	1733	37553	35820	-	*Vigna radiata*
	nad1a	383	82568	82185	-	*Vigna radiata*
	nad2a	152	176155	176307	+	*Vigna radiata*
	nad2b	401	177548	177949	+	*Vigna radiata*
	nad5a	236	45369	45605	+	*Vigna radiata*
	nad5b	1259	46387	47646	+	*Vigna radiata*
	nad6	614	79543	78929	-	*Vigna radiata*
	nad7a	161	165044	164883	-	*Vigna radiata*
	nad7b	101	164033	163932	-	*Cucurbita pepo*
	nad7c	464	162595	162131	-	*Vigna radiata*
	nad7d	245	161081	160836	-	*Vigna radiata*
	nad7e	323	159026	158703	-	*Vigna radiata*
	rps1	620	44567	45187	+	*Carica papaya*
	rps12	374	108970	109344	+	*Vigna radiata*
	rps4	194	60217	60023	-	*Vigna radiata*
	rps7	74	34404	34330	-	*Citrullus lanatus*
	trnM	73	190693	190766	+	*Nicotiana tabacum*
	trnF	73	194813	194886	+	*Vigna radiata*
	trnP	74	195144	195218	+	*Cucumis sativus*
	trnS	87	194364	194451	+	*Zea perennis*
Scaffold13	*atp1*	1511	14406	12895	-	*Vigna radiata*
	atp8	479	24608	25087	+	*Vigna radiata*
	ccmB	59	22783	22842	+	*Vigna radiata*
	nad5a	107	12862	12755	-	*Arabidopsis thaliana*
	trnC	72	31587	31659	+	*Vigna radiata*
	trnH	75	33343	33418	-	*Beta vulgaris*
	trnM	73	24359	24432	+	*Nicotiana tabacum*

Scaffold17	*atp1*	476	158466	157990	-	*Nicotiana tabacum*
	atp4	569	32026	32595	+	*Vigna radiata*
	atp9	65	13907	13842	-	*Beta vulgaris*
	cob	1172	56638	57810	+	*Vigna radiata*
	cox1	1580	37187	38767	+	*Vigna radiata*
	matR	2002	109758	111760	+	*Vigna radiata*
	mttB	722	133343	132621	-	*Citrullus lanatus*
	nad1b	92	40244	40336	+	*Vigna radiata*
	nad1c	194	41196	41390	+	*Vigna radiata*
	nad1e	254	112252	112506	+	*Vigna radiata*
	nad2c	158	73097	72939	-	*Vigna radiata*
	nad2d	638	70419	69781	-	*Vigna radiata*
	nad2e	185	68357	68172	-	*Vigna radiata*
	nad4a	458	88644	89102	+	*Vigna radiata*
	nad4b	584	90493	91077	+	*Vigna radiata*
	nad4c	419	94122	94541	+	*Vigna radiata*
	nad4d	86	97105	97191	+	*Vigna radiata*
	nad5c	395	50665	51060	+	*Vigna radiata*
	nad5d	137	51973	52110	+	*Vigna radiata*
	rpl16	535	46215	46750	+	*Arabidopsis thaliana*
	rpl5	554	54859	55413	+	*Vigna radiata*
	rps10a	248	33793	34041	+	*Vigna radiata*
	rps10b	119	36865	36984	+	*Vigna radiata*
	rps14	299	55424	55723	+	*Vigna radiata*
	rps19b	119	42756	42875	+	*Cucurbita pepo*
	rps3a	74	42892	42966	+	*Vigna radiata*
	rps3b	1583	44729	46312	+	*Vigna radiata*
	sdh4	62	64990	64928	-	*Citrullus lanatus*
	trnM	72	115524	115596	+	*Vigna radiata*
	trnC	70	120961	121031	-	*Cucumis sativus,*
	trnQ	71	118507	118578	-	*Arabidopsis thaliana*
	trnE	71	114612	114683	+	*Vigna radiata*
	trnK	72	159140	159212	-	*Citrullus lanatus*
Scaffold101	*rps19a*	77	27798	27875	+	*Carica papaya*
	trnN	71	31578	31649	+	*Beta vulgaris*
	trnG	72	28246	28318	+	*Beta vulgaris*
	trnK	72	8709	8781	+	*Vigna radiata*
	trnY	82	32021	32103	+	*Vigna radiata*

Table S4: Gene annotation of scaffolds derived from mitochondrial sequences of ICPW 29.

ScaffoldID	Genes	Size	Coordinates		Strand	Organism with top hit
			From	To		
Scaffold1	*atp1*	1511	98948	97437	-	*Vigna radiata*
	ccmB	617	77628	78245	+	*Vigna radiata*
	cob	1172	148380	149552	+	*Vigna radiata*
	cox3	794	108456	109250	+	*Vigna radiata*
	matR	2003	202103	204106	+	*Vigna radiata*
	mttB	722	225652	224930	-	*Citrullus lanatus*
	nad1a	383	78921	79304	+	*Vigna radiata*
	nad1d	95	201394	201489	+	*Vigna radiata*
	nad1e	254	204598	204852	+	*Vigna radiata*
	nad2c	158	164840	164682	-	*Vigna radiata*
	nad2d	638	162161	161523	-	*Vigna radiata*
	nad2e	185	160099	159914	-	*Vigna radiata*
	nad3	353	52916	52563	-	*Vigna radiata*
	nad4a	458	180979	181437	+	*Vigna radiata*
	nad4b	584	182829	183413	+	*Vigna radiata*
	nad4c	419	186460	186879	+	*Vigna radiata*
	nad4d	86	189445	189531	+	*Vigna radiata*
	nad5c	395	142404	142799	+	*Vigna radiata*
	nad5d	137	143712	143849	+	*Vigna radiata*
	nad6	614	81947	82561	-	*Vigna radiata*
	rpl5	554	146598	147152	+	*Vigna radiata*
	rps12	374	52511	52137	+	*Vigna radiata*
	rps14	299	147163	147462	+	*Vigna radiata*
	sdh4	239	109181	109420	+	*Citrullus lanatus*
	trnD	73	179499	179572	-	*Nicotiana tabacum*
	trnC	70	213262	213332	+	*Vigna radiata*
	trnQ	71	210808	210879	-	*Cucumis sativus*
	trnE	71	206958	207029	+	*Arabidopsis thaliana*
	trnM	72	41732	41804	+	*Nicotiana tabacum*
	trnM	72	207870	207942	+	*Citrullus lanatus*
	trnF	73	241150	241223	-	*Vigna radiata*
	trnP	74	240817	240891	-	*Cucumis sativus*
	trnS	87	241585	241672	-	*Zea perennis*
Scaffold2	*ccmC*	737	10172	9435	-	*Vigna radiata*
	ccmFn	1733	12593	10860	-	*Vigna radiata*
	nad5b	1193	21696	22889	+	*Vigna radiata*

	nad7a	161	142571	142410	-	*Vigna radiata*
	nad7b	101	141560	141459	-	*Cucurbita pepo*
	nad7c	464	140122	139658	-	*Vigna radiata*
	nad7d	245	138608	138363	-	*Vigna radiata*
	nad7e	323	136551	136228	-	*Vigna radiata*
	nad9	491	93404	93895	+	*Vigna radiata*
	rps1	620	19608	20228	+	*Carica papaya*
	rps12	149	42211	42360	+	*Vigna radiata*
	rps14	68	36215	36147	-	*Vigna radiata*
	rps19a	77	64757	64680	-	*Carica papaya*
	rps19b	59	156654	156713	+	*Carica papaya*
	rps4	1037	35953	34916	-	*Vigna radiata*
	rps7	74	9445	9371	-	*Citrullus lanatus*
	trnN	71	60615	60686	-	*Beta vulgaris*
	trnC	72	151173	151245	-	*Vignaradiata*
	trnG	72	64237	64309	-	*Beta vulgaris*
	trnH	75	149413	149488	-	*Beta vulgaris*
	trnK	72	84781	84853	-	*Vigna radiata*
	trnW-cp	73	104998	105071	-	*Vigna_radiata*
	trnY	82	60161	60243	-	*Vigna_radiata*
Scaffold3	*atp4*	569	8368	7799	-	*Vigna radiata*
	atp6	1154	61500	60346	-	*Nicotiana tabacum*
	atp8	479	90319	90798	+	*Vigna radiata*
	atp9	65	27204	27269	+	*Beta vulgaris*
	ccmFCa	779	45898	46677	+	*Vigna radiata*
	ccmFCb	566	50179	50745	+	*Vigna radiata*
	cox1	1580	3207	1627	-	*Vigna radiata*
	nad1b	92	149	57	-	*Vigna radiata*
	nad2a	152	75639	75791	+	*Vigna radiata*
	nad2b	401	77032	77433	+	*Vigna radiata*
	nad4L	296	8863	8567	-	*Vigna radiata*
	rps10a	248	6601	6353	-	*Vigna radiata*
	rps10b	119	3529	3410	-	*Vigna radiata*
	trnM					*Nicotiana tabacum*
Scaffold9	*nad1c*	194	847	1041	+	*Vigna radiata*
	rpl16	511	5890	6401	+	*Arabidopsis thaliana*
	rps19b	119	2407	2526	+	*Cucurbita pepo*
	rps3a	74	2543	2617	+	*Vigna radiata*
	rps3b	1583	4380	5963	+	*Vigna radiata*

Table S5: Gene annotation of scaffolds derived from mitochondrial sequences of ICPB 2039.

ScaffoldID	Genes	Size	Coordinates		Strand	Organism with top hit
			From	To		
Scaffold10	*nad2a*	152	2143	1991	-	*Vigna radiata*
	nad2b	401	750	349	-	*Vigna radiata*
	rps10	47	2296	2249	-	*Vigna radiata*
Scaffold13	*nad3*	77	180	103	-	*Vigna radiata*
	rps12	173	6067	6240	+	*Brassica napus*
	rps14	68	96	28	-	*Vigna radiata*
Scaffold1	*nad3*	353	14036	13683	-	*Vigna radiata*
	rps12	374	13631	13257	-	*Vigna radiata*
Scaffold30	*rps19*	77	4068	3991	-	*Carica papaya*
	trnN	71	216	287	-	*Beta vulgaris*
	trnG	71	3548	3619	-	*Beta vulgaris*
Scaffold31	*ccmFn*	455	458	3	-	*Vigna radiata*
Scaffold33	*atp9*	221	3980	3759	-	*Vigna radiata*
Scaffold34	*matR*	164	1616	1452	-	*Vigna radiata*
	nad1	254	960	706	-	*Vigna radiata*
	trnE	70	4851	4921	-	*Arabidopsis thaliana*
	trnM	72	3938	4010	-	*Citrullus lanatus*
Scaffold3	*ccmB*	59	1956	2015	+	*Vigna radiata*
	cox3	794	7881	7087	-	*Vigna radiata*
	sdh4	323	7156	6833	-	*Citrullus lanatus*
Scaffold45	*nad4*	419	2112	1693	-	*Vigna radiata*
Scaffold49	*nad2c*	158	1280	1438	+	*Vigna radiata*
Scaffold7	*nad9*	570	15190	14620	-	*Nicotiana tabacum*
Scaffold16	*trnK*	72	1549	1621	+	*Vigna radiata*
Scaffold17	*trnW-cp*	73	7159	7232	+	*Vigna radiata*
Scaffold25	trnM	71	3331	3402	+	*Zea mays*
Scaffold36	*trnW-cp*	73	7159	7232	+	*Vigna radiata*
Scaffold5	*trnF*	73	17592	17665	-	*Vigna radiata*
	trnP	74	17260	17334	-	*Cucumis sativus*
	trnS	87	18027	18114	-	*Zea perennis*
Scaffold6	*trnC*	70	10058	10128	+	*Vigna radiata*
	trnQ	71	13392	13463	-	*Cucumis sativus*

Table S6: Genomic rearrangements observed in ICPA 2039 while comparing with mitochondrial sequences of ICPB 2039, ICPW 29 and ICPH 2433 separately.

Rearrangement Regions		ICPA 2039/ ICPB 2039	ICPA 2039/ ICPW 29	ICPA 2039/ ICPH 2433
From	To			
3441	7072	✓	-	-
15709	18693	✓	-	-
34909	165239	✓	-	-
72614	403154	✓	✓	-
107485	219108	-	✓	-
143366	144892	✓	-	-
164987	165000	-	✓	-
170658	225317	-	✓	-
187564	399514	✓	-	-
191084	489849	-	✓	-
193458	195972	✓	-	-
249675	263641	✓	✓	-
251365	253166	✓	-	-
252598	252974	✓	-	-
262266	401325	✓	✓	-
262352	263295	✓	-	-
262339	401289	-	✓	-
263124	470227	✓	✓	-
275554	471358	✓	-	-
276013	470845	✓	-	-
298472	298979	✓	-	-
306210	307224	✓	-	-
342909	344446	✓	-	-
379049	382176	✓	-	-
433127	484083	✓	-	-
443386	444100	✓	-	-
446611	451191	✓	-	-

Table S7: No coverage regions observed in ICPA 2039 while comparing with mitochondrial sequences of ICPB 2039, ICPW 29 and ICPH 2433 separately.

**No Coverage Regions**			**ICPA 2039 / ICPB 2039**	**ICPA 2039/ ICPW 29**	**ICPA 2039/ ICPH 2433**
**From**	**To**	**Length**			
489850	499083	9234	✓	-	-
476053	484470	8418	✓	-	-
212270	219837	7568	✓	-	-
165134	170282	5149	✓	-	-
419186	424114	4929	✓	-	-
257348	262265	4918	✓	-	-
531943	536388	4446	✓	-	-
265782	269725	3944	✓	-	-
69876	72608	2733	✓	-	-
411099	413552	2454	✓	-	-
309903	312076	2174	✓	-	-
430061	432077	2017	✓	-	-
157104	158668	1565	✓	-	-
81894	83428	1535	✓	-	-
324064	325537	1474	✓	-	-
263655	265027	1373	✓	-	-
222478	223535	1058	✓	-	-
489860	499011	9152	-	✓	-
476087	484001	7915	-	✓	-
212269	219101	6833	-	✓	-
257348	262265	4918	-	✓	-
419548	424117	4570	-	✓	-
166121	170233	4113	-	✓	-
265782	269725	3944	-	✓	-
69913	72520	2608	-	✓	-
411099	413510	2412	-	✓	-
430061	432077	2017	-	✓	-
310202	312008	1807	-	✓	-
263655	265107	1453	-	✓	-
217615	218657	1043	-	-	✓
257653	261955	4303	-	-	✓

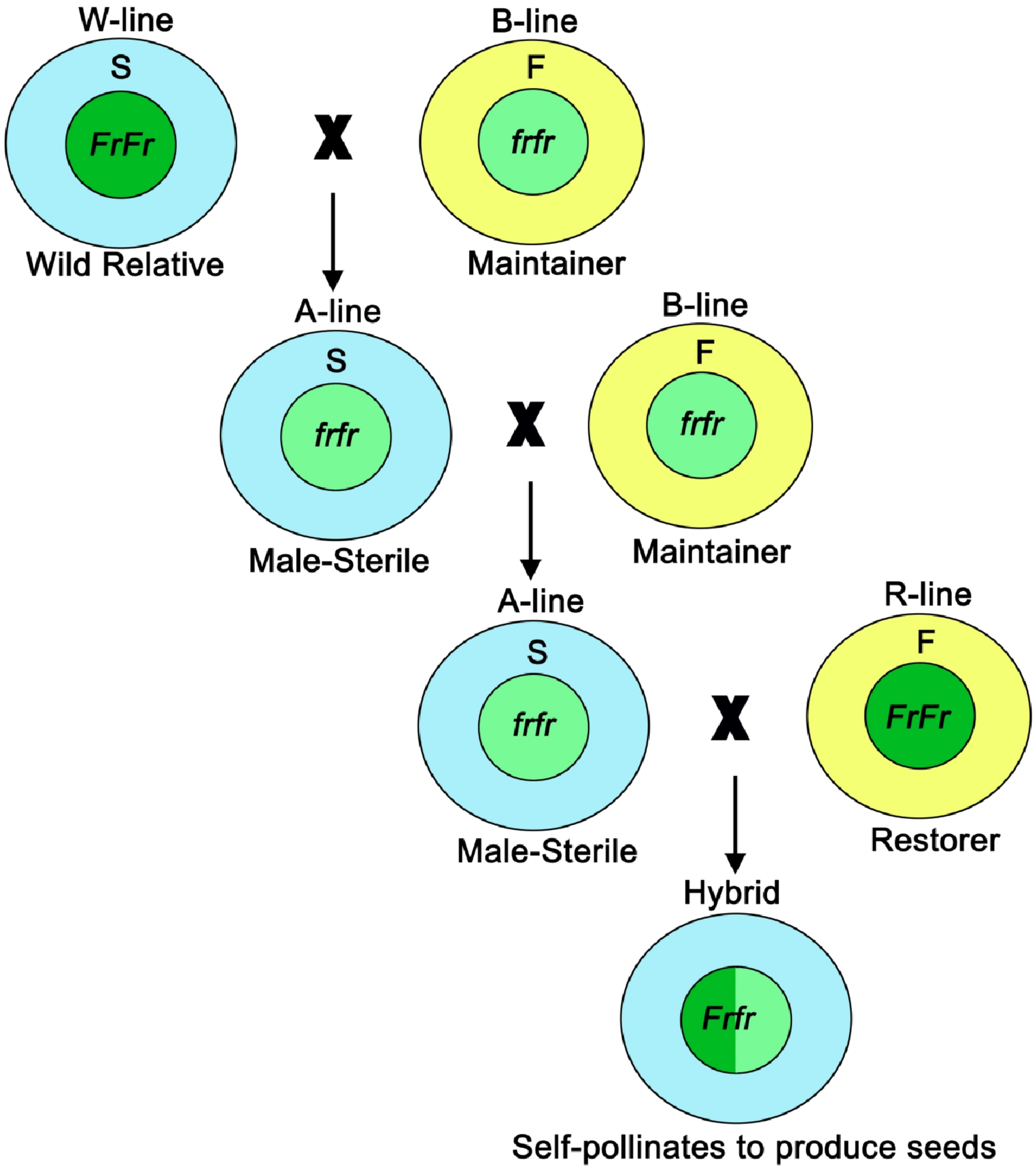

Supplementary Figure S1. A general methodology for the production of commercial hybrids by using cytoplasmic male sterility system.

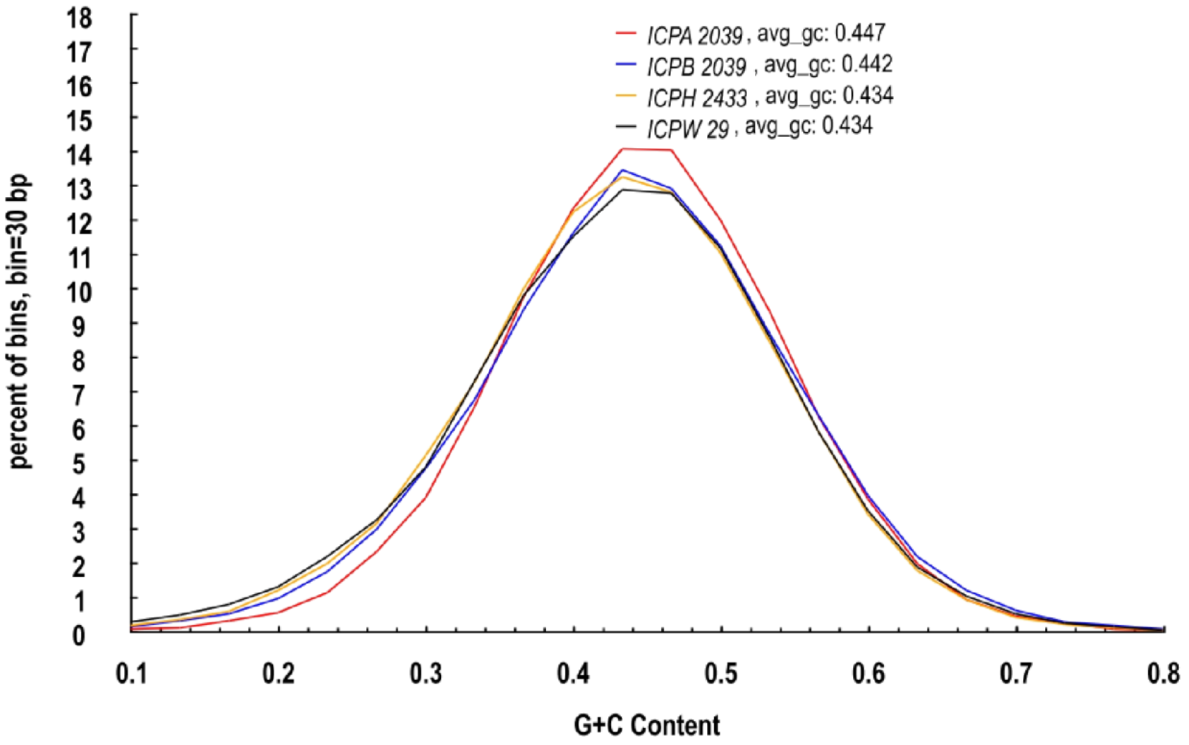

Supplemental Figure S2. Graph represents GC content distribution in mitochondrial genomes of ICPA 2039, ICPB 2039, ICPH 2433 and ICPW 29 in red, blue, yellow and black colours respectively.

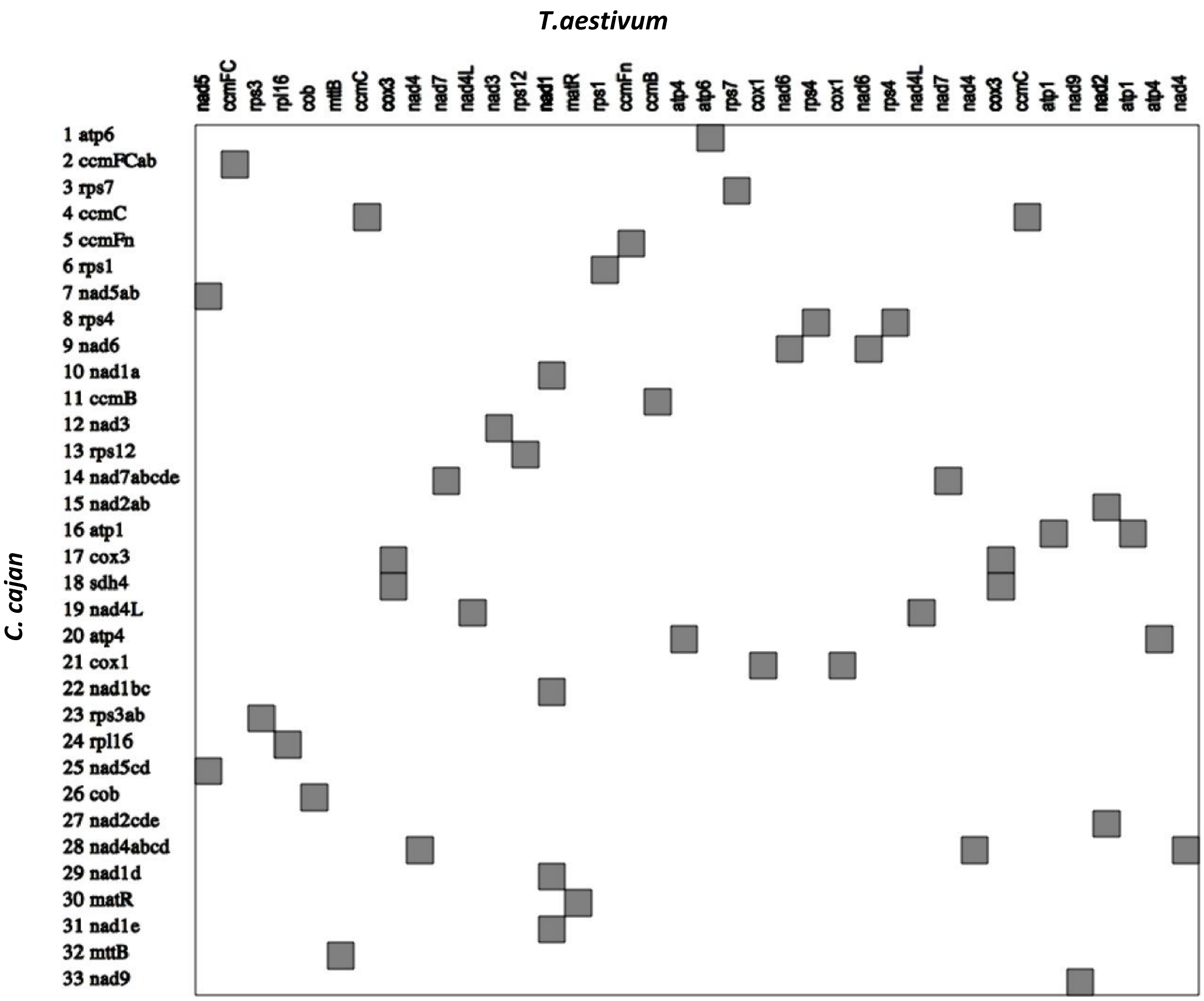

Supplemental Figure S3. Correlation of gene order between the mitochondrial gene maps of *Cajanus cajan* and *Triticium aestivum*.

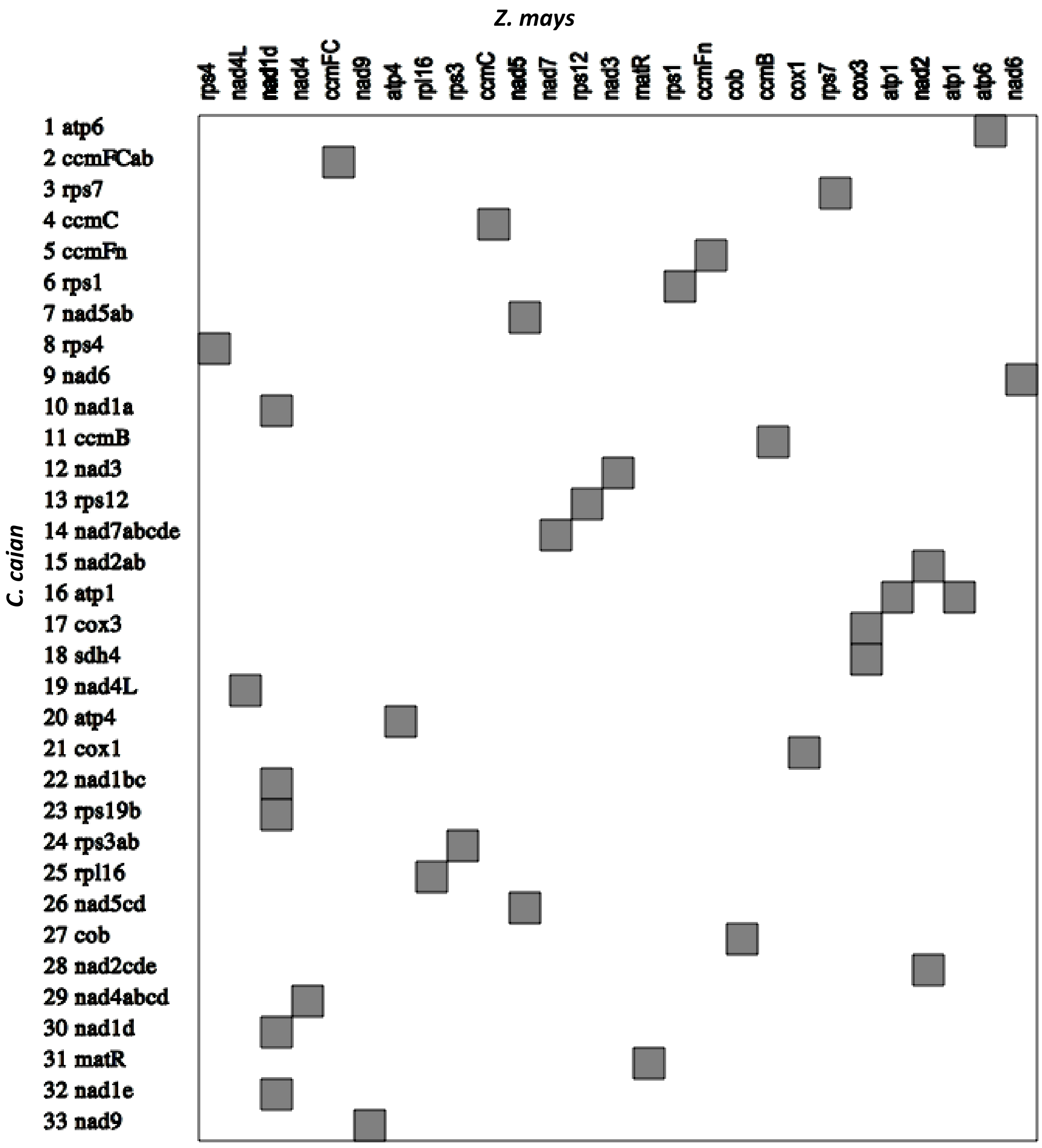

Supplemental Figure S4. Correlation of gene order between the mitochondrial gene maps of *Cajanus cajan* and *Zea mays*.

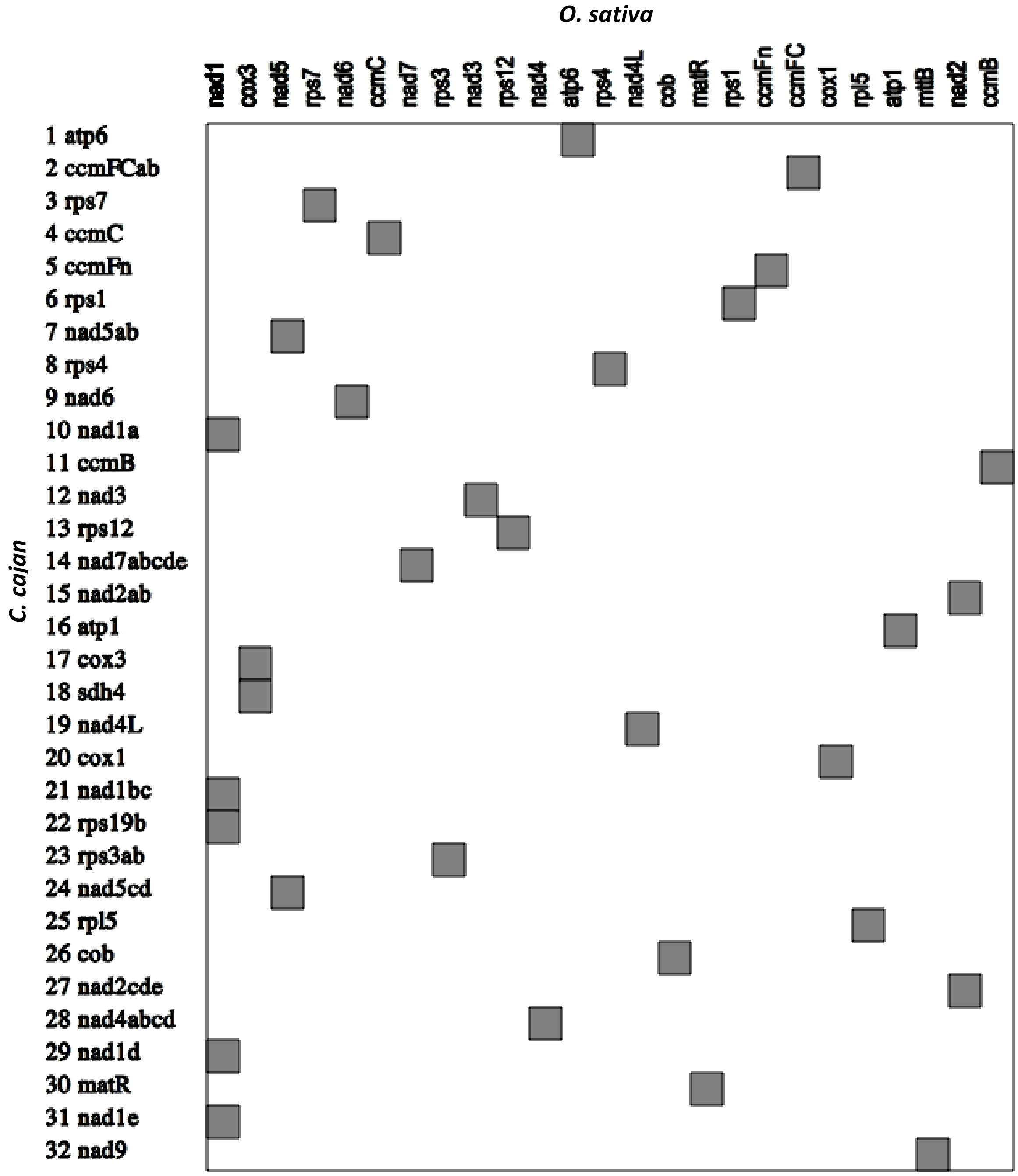

Supplemental Figure S5. Correlation of gene order between the mitochondrial gene maps of *Cajanus cajan* and *Oryza sativa*.

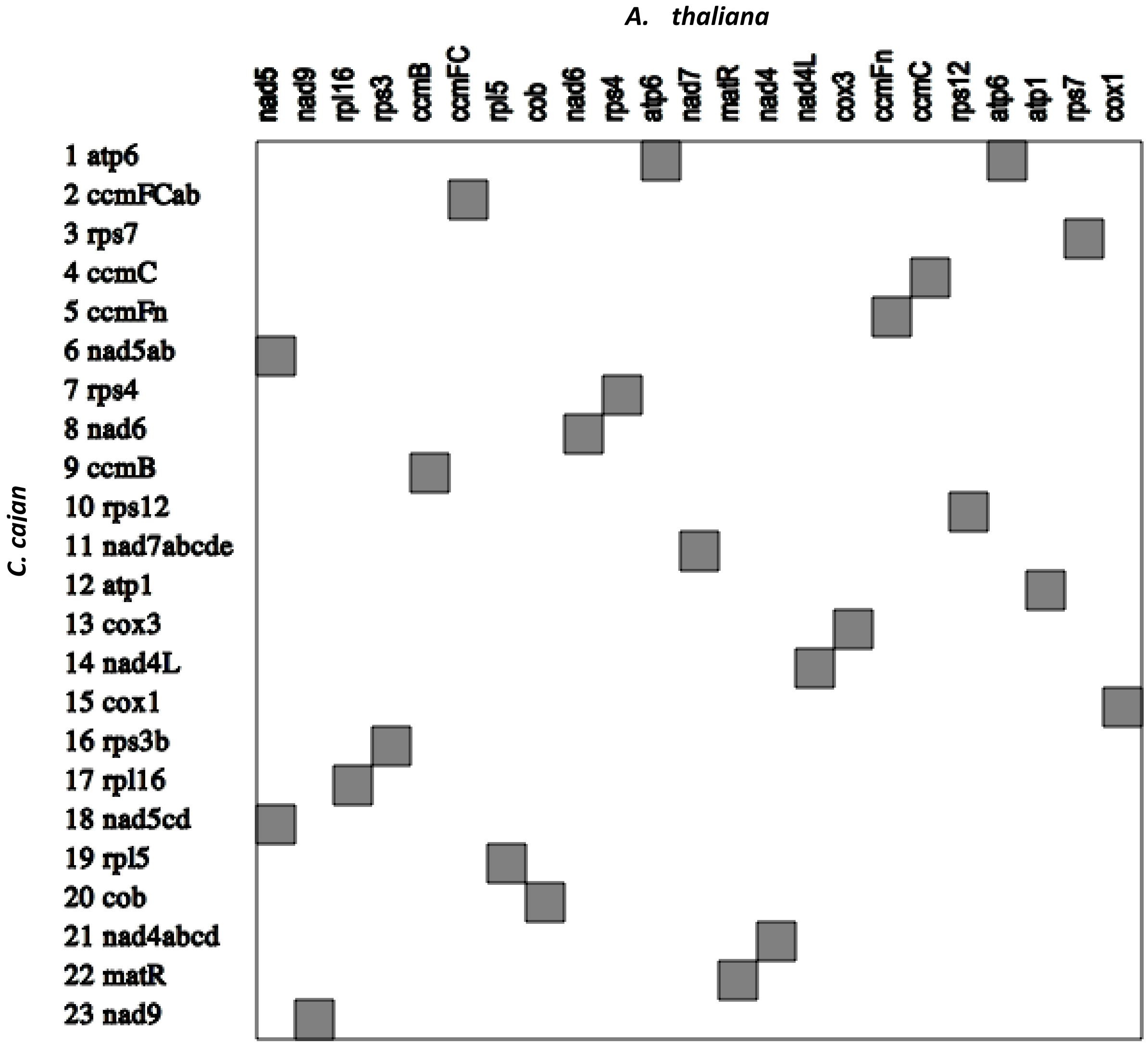

Supplemental Figure S6. Correlation of gene order between the mitochondrial gene maps of *Cajanus cajan* and *Arabidopsis thaliana*.

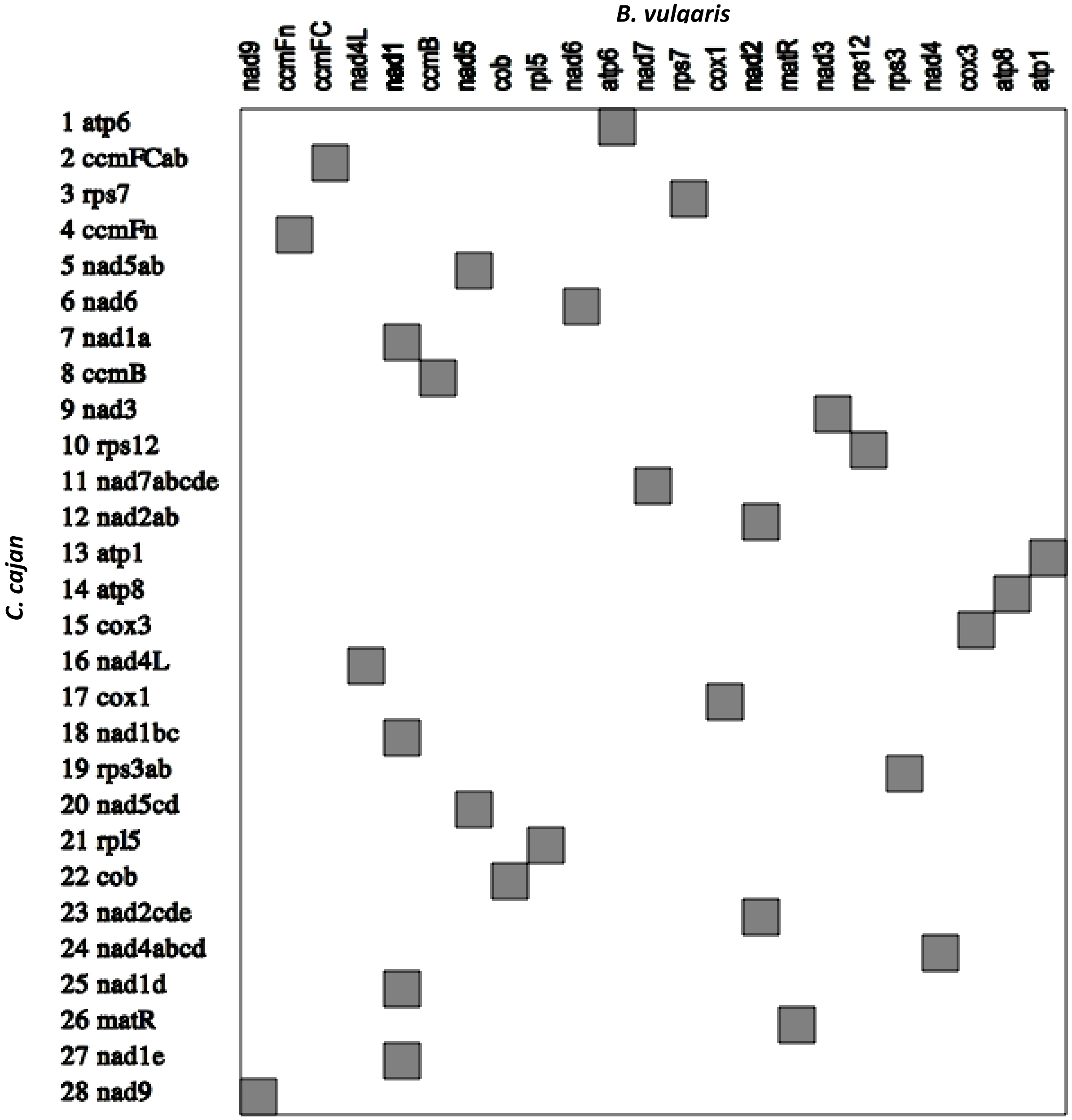

Supplemental Figure S7. Correlation of gene order between the mitochondrial gene maps of *Cajanus cajan* and *Beta vulgaris*.

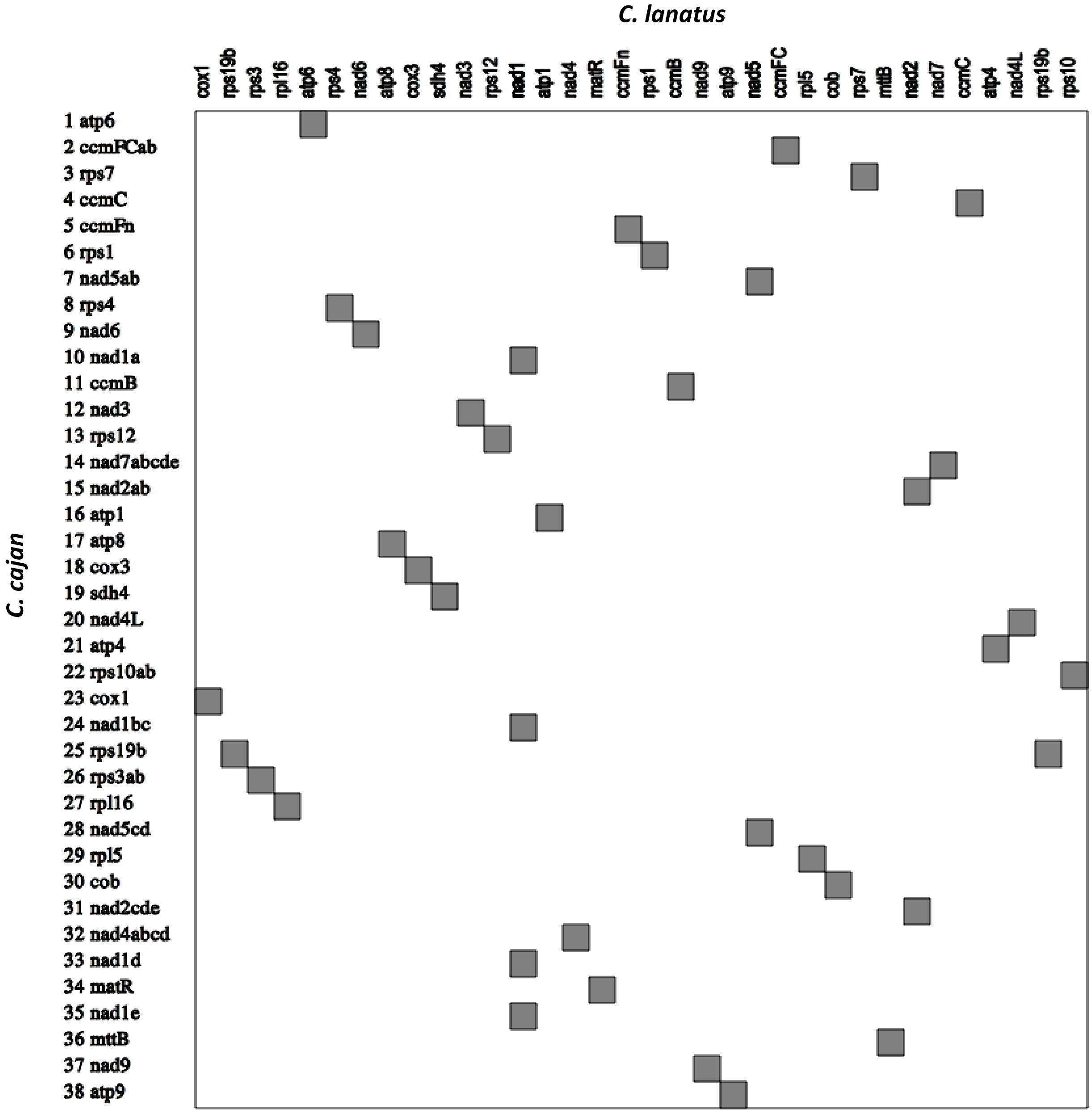

Supplemental Figure S8. Correlation of gene order between the mitochondrial gene maps of *Cajanus cajan* and *Citrullus lanatus*.

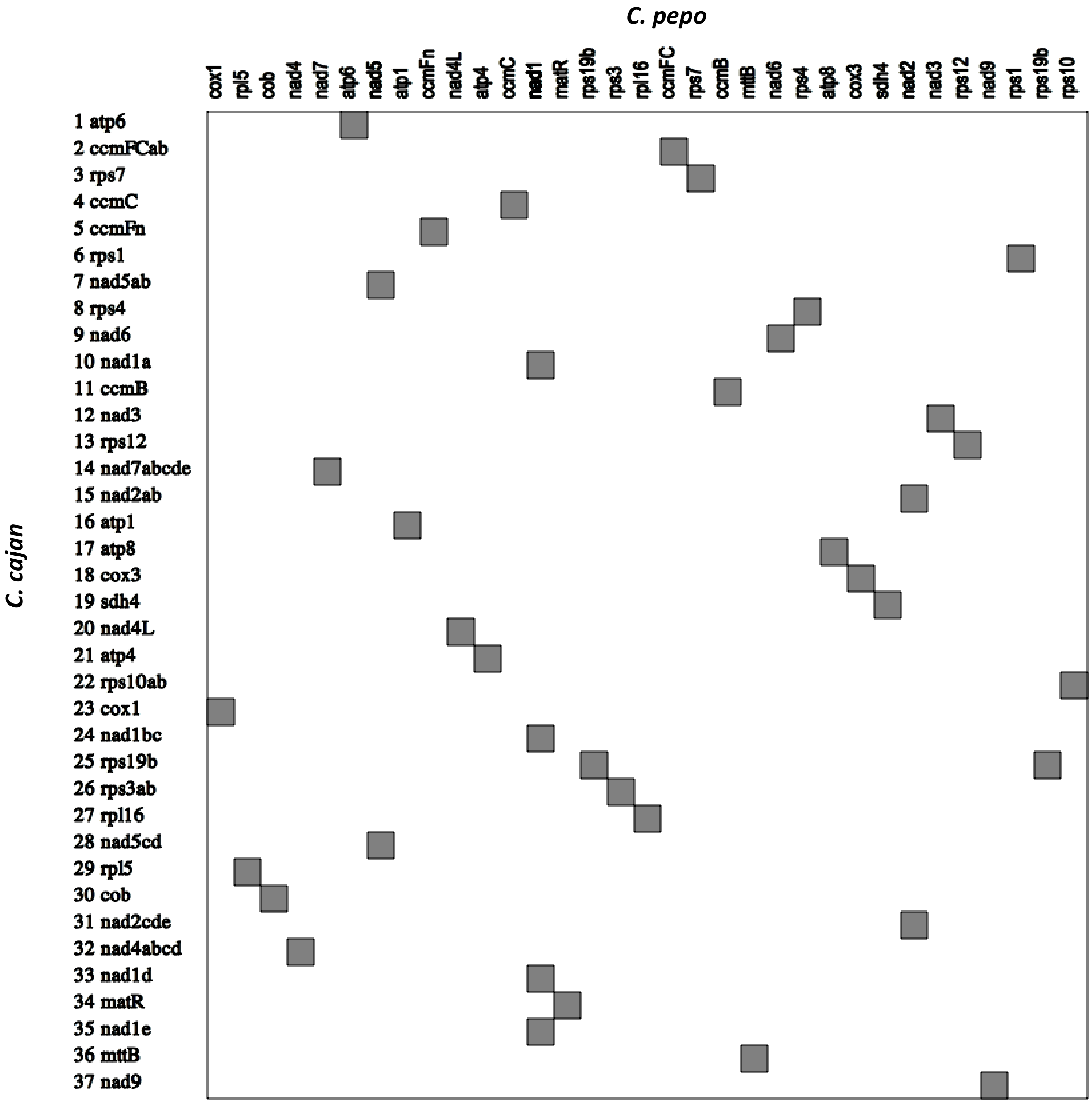

Supplemental Figure S9. Correlation of gene order between the mitochondrial gene maps of *Cajanus cajan* and *Cucurbita pepo*.

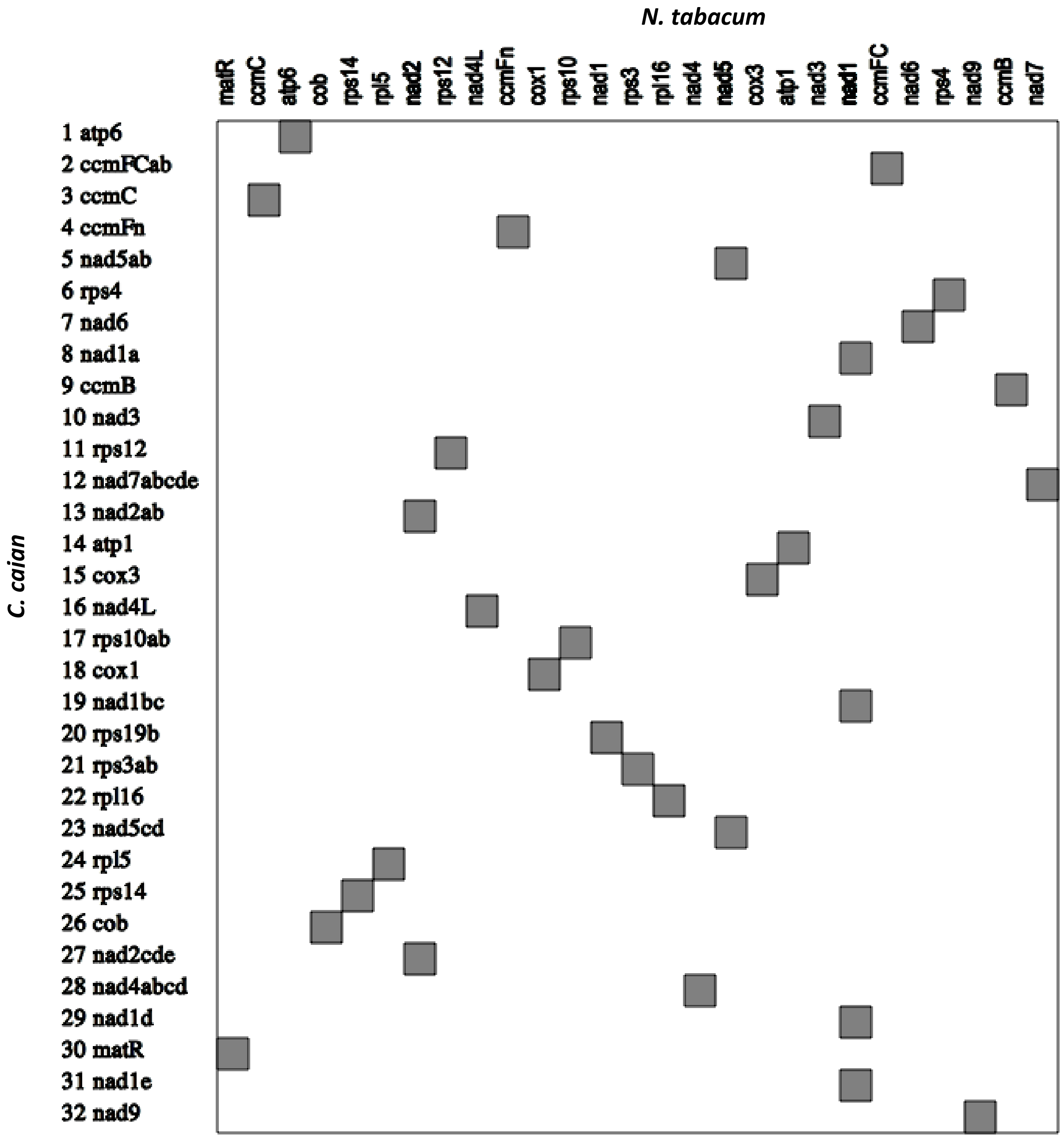

Supplemental Figure S10. Correlation of gene order between the mitochondrial gene maps of *Cajanus cajan* and *Nicotina tabacum*.

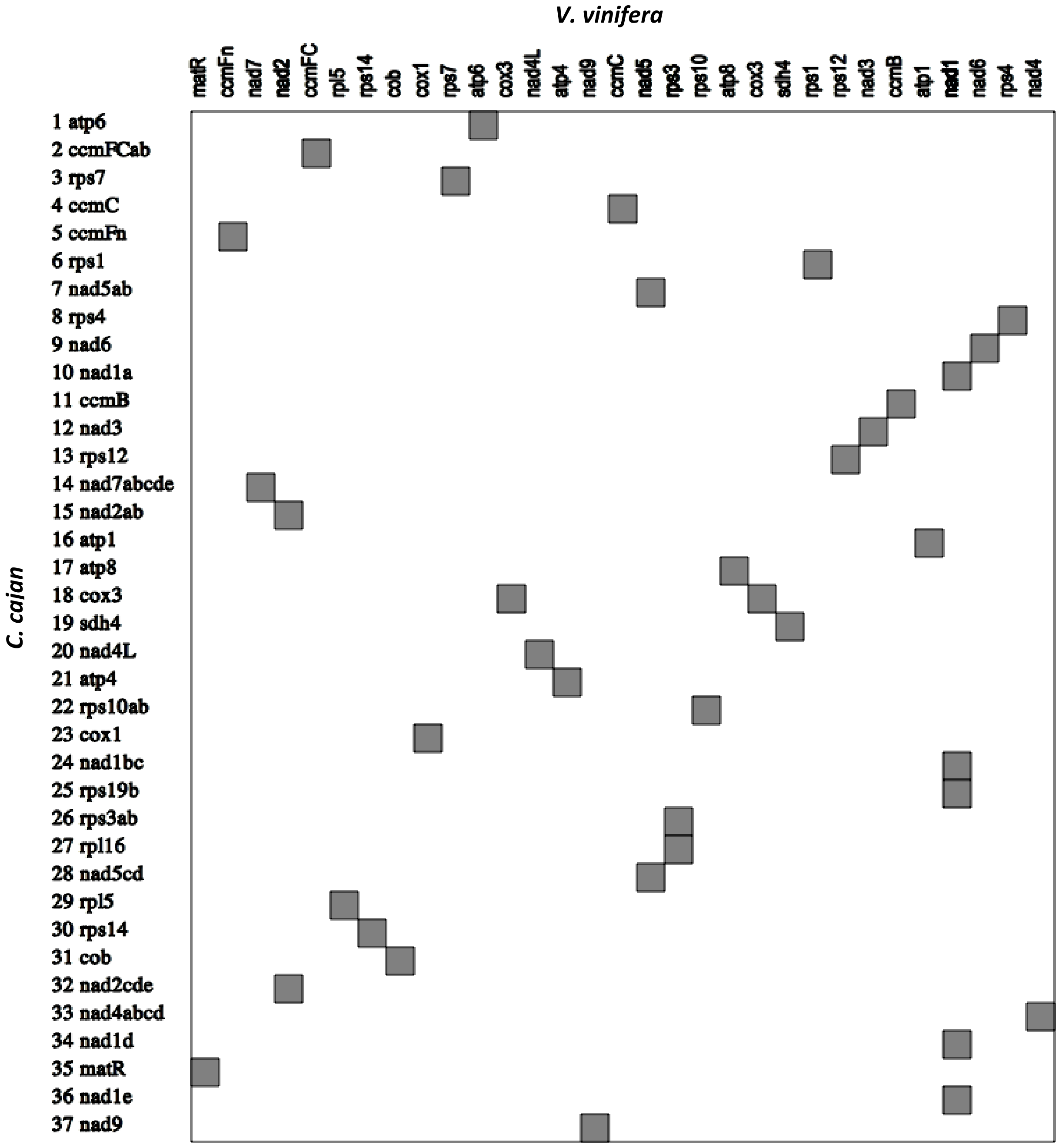

Supplemental Figure S11. Correlation of gene order between the mitochondrial gene maps of *Cajanus cajan* and *Vitis vinifera*.

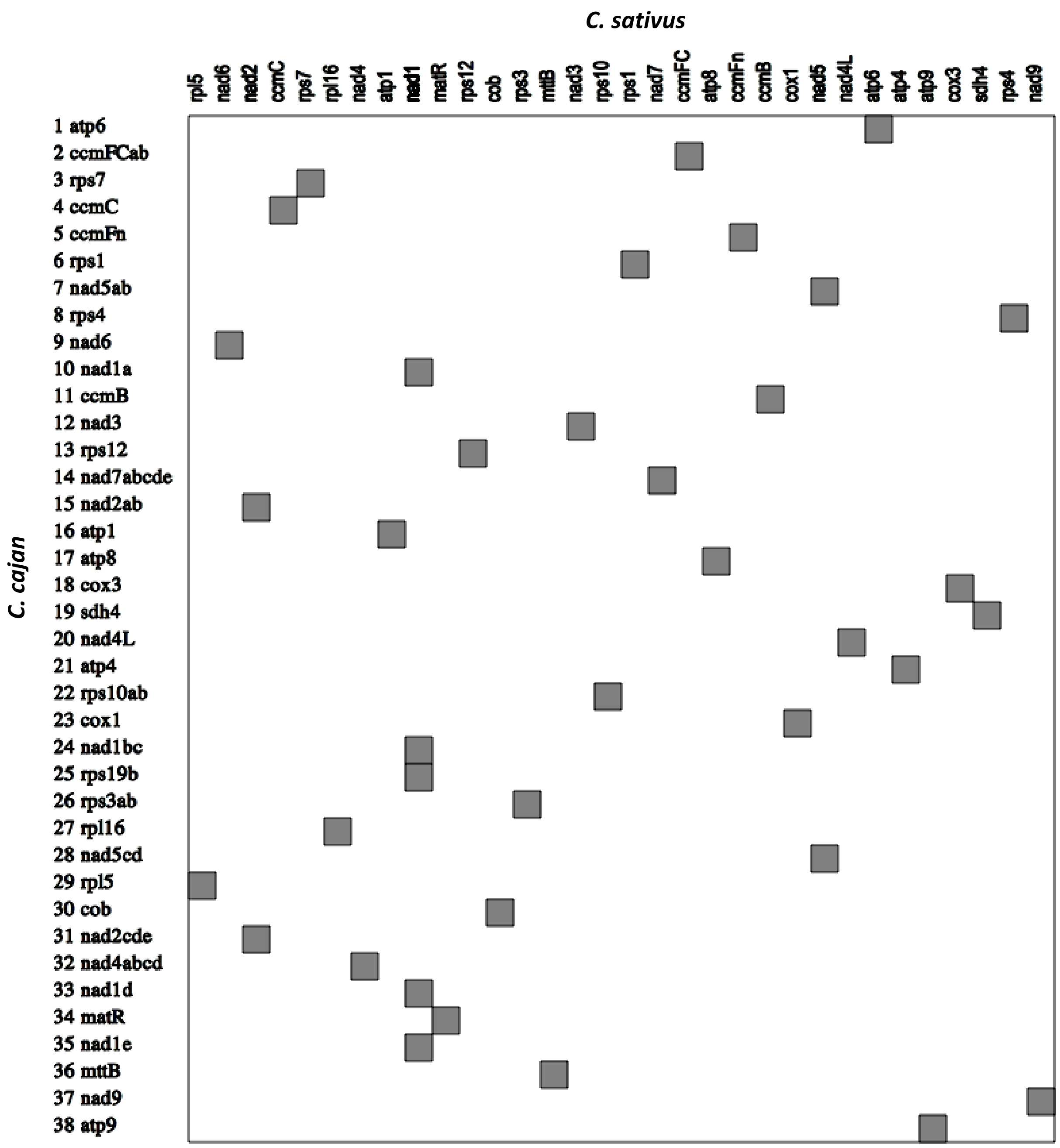

Supplemental Figure S12. Correlation of gene order between the mitochondrial gene maps of *Cajanus cajan* and *Cucumis sativus*.

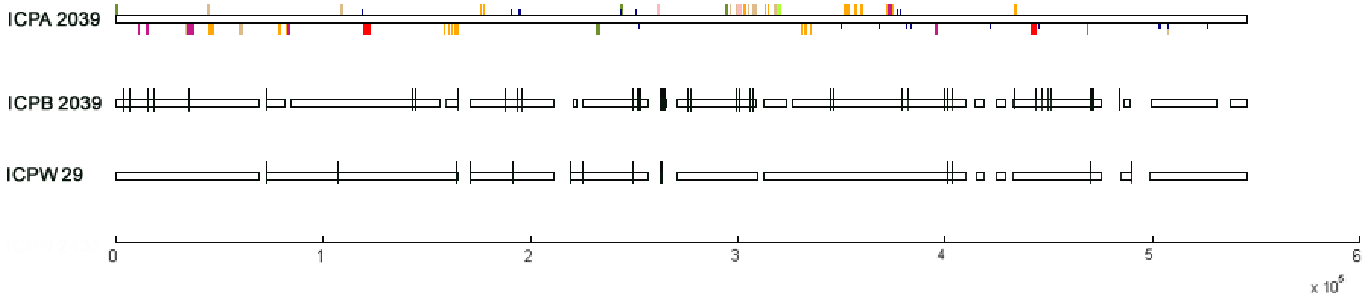

Supplemental Figure S13. Output of GS Reference Mapper 2.5. software which represents rearrangements and no-coverage regions identified after aligning additional sequence reads (as datasets were generated a second time) of ICPB 2039 and ICPW 29 to the final assembly of ICPA 2039.
